# Demethylation of EHMT1/GLP Protein Reprograms Its Transcriptional Activity and Promotes Prostate Cancer Progression

**DOI:** 10.1158/2767-9764.CRC-23-0208

**Published:** 2023-08-31

**Authors:** Anna Besschetnova, Wanting Han, Mingyu Liu, Yanfei Gao, Muqing Li, Zifeng Wang, Maryam Labaf, Susan Patalano, Kavita Venkataramani, Rachel E. Muriph, Jill A. Macoska, Kellee R. Siegfried, Jason Evans, Steven P. Balk, Shuai Gao, Dong Han, Changmeng Cai

**Affiliations:** 1 Center for Personalized Cancer Therapy, University of Massachusetts Boston, Boston, Massachusetts.; 2 Department of Biology, University of Massachusetts Boston, Boston, Massachusetts.; 3 Human Biology Division, Fred Hutchinson Cancer Center, Seattle, Washington.; 4 Department of Orthopedics, The Second Affiliated Hospital, Chongqing Medical University, Chongqing, P.R. China.; 5 Department of Mathematics, University of Massachusetts Boston, Boston, Massachusetts.; 6 Department of Chemistry, University of Massachusetts Boston, Boston, Massachusetts.; 7 Hematology-Oncology Division, Department of Medicine, Beth Israel Deaconess Medical Center and Harvard Medical School, Boston, Massachusetts.; 8 Department of Cell Biology and Anatomy, New York Medical College, Valhalla, New York.; 9 Department of Biochemistry and Molecular Biology, New York Medical College, Valhalla, New York.

## Abstract

Epigenetic reprogramming, mediated by genomic alterations and dysregulation of histone reader and writer proteins, plays a critical role in driving prostate cancer progression and treatment resistance. However, the specific function and regulation of EHMT1 (also known as GLP) and EHMT2 (also known as G9A), well-known histone 3 lysine 9 methyltransferases, in prostate cancer progression remain poorly understood. Through comprehensive investigations, we discovered that both EHMT1 and EHMT2 proteins have the ability to activate oncogenic transcription programs in prostate cancer cells. Silencing EHMT1/2 or targeting their enzymatic activity with small-molecule inhibitors can markedly decrease prostate cancer cell proliferation and metastasis *in vitro* and *in vivo*. In-depth analysis of posttranslational modifications of EHMT1 protein revealed the presence of methylation at lysine 450 and 451 residues in multiple prostate cancer models. Notably, we found that lysine 450 can be demethylated by LSD1. Strikingly, concurrent demethylation of both lysine residues resulted in a rapid and profound expansion of EHMT1’s chromatin binding capacity, enabling EHMT1 to reprogram the transcription networks in prostate cancer cells and activate oncogenic signaling pathways. Overall, our studies provide valuable molecular insights into the activity and function of EHMT proteins during prostate cancer progression. Moreover, we propose that the dual-lysine demethylation of EHMT1 acts as a critical molecular switch, triggering the induction of oncogenic transcriptional reprogramming in prostate cancer cells. These findings highlight the potential of targeting EHMT1/2 and their demethylation processes as promising therapeutic strategies for combating prostate cancer progression and overcoming treatment resistance.

**Significance:**

In this study, we demonstrate that EHMT1 and EHMT2 proteins drive prostate cancer development by transcriptionally activating multiple oncogenic pathways. Mechanistically, the chromatin binding of EHMT1 is significantly expanded through demethylation of both lysine 450 and 451 residues, which can serve as a critical molecular switch to induce oncogenic transcriptional reprogramming in prostate cancer cells.

## Introduction

Prostate cancer initially responds to androgen deprivation therapies (ADT), which inhibit the transcriptional activity of the androgen receptor (AR). However, the emergence of castration-resistant prostate cancer (CRPC), a more aggressive metastatic form, poses a significant challenge (1). Second-generation ADTs, such as enzalutamide, apalutamide, and abiraterone, have been employed to treat CRPC by further blocking AR activity or androgen biosynthesis (2–4). Nonetheless, tumor cells can evade these treatments through various mechanisms, including epigenetic reprogramming of transcriptional networks mediated by alterations in chromatin reader/writer proteins (5). Consequently, targeting these epigenetic factors has emerged as a new promising strategy for CRPC treatment (6–9). Lysine methylations on histone tails play critical roles in gene transcription, with dynamic regulation by the lysine methyltransferase family proteins (KMT) and lysine demethylase proteins (KDM; ref. 10). Dysregulation of KMTs/KDMs is frequently associated with diseases, including cancer (11). In prostate cancer, mutations of *KMT2* genes (also known as mixed lineage leukemia family methylates) and dysregulation of *EZH2* (enhancer of zeste 2 polycomb repressive complex 2 subunit) and *LSD1* (lysine-specific demethylase 1, also known as *KDM1A*) are commonly observed and further enriched in CRPC (5, 12).

EHMT1 (euchromatin histone methyltransferase 1, also known as GLP) and EHMT2 (euchromatin histone methyltransferase 2, also known as G9a) are well-known SET domain-containing methyltransferases that methylate histone 3 lysine 9 (H3K9), contributing to gene transcription repression (13). Both EHMT1 and EHMT2 are aberrantly expressed in various cancers and associated with epithelial–mesenchymal transition (EMT), cancer stemness, and metastasis (14–17). EHMT1 forms heterodimer with EHMT2 to methylate H3K9, generating monomethylated or dimethylated H3K9 (13). In addition, both proteins have been reported to methylate non-histone proteins (18, 19). EHMTs, along with LSD1 (an H3K4 demethylase), are components of the REST complex, which silences neuronal pathway genes in non-neuronal cells (20). Recent studies have implicated EHMT1 and EHMT2 as critical epigenetic drivers in prostate cancer. One study suggests that EHMT1 protein stabilization in *SPOP* mutation prostate cancer leads to increased DNA methylation (9). Another study proposes that EHMT1, along with other H3K9 methyltransferases, drives prostate cancer resistance to ADTs by enabling transcriptional silencing of retroelements (21). In addition, a recently published study reveals the oncogenic function of EHMT2 in mediating the function of the PRC2 (polycomb repressive complexes 2) complex in prostate cancer cells (22). However, there is still an urgent need to comprehensively understand the molecular function and regulation of EHMTs in prostate cancer cells.

In this study, we characterized the transcriptional targets of EHMT proteins in prostate cancer models. Our findings demonstrate that both EHMT1 and EHMT2 can activate oncogenic programs, including E2F and MYC signaling. Silencing EHMT1 or EHMT2 effectively suppresses prostate cancer cell proliferation and migration. Furthermore, we tested several dual EHMT1/2 inhibitors in prostate cancer models, and UNC0642 (23) stood out as a potential inhibitor that decreases prostate cancer tumor growth and metastasis *in vivo*. Subsequently, we conducted proteomic analyses to identify functionally significant posttranslational modifications of EHMT1. Our investigation revealed dual-lysine K450/K451 methylations at the EHMT1 protein, with K450 being demethylated by LSD1. While demethylation of K450 or K451 alone did not significantly impact the chromatin activity of EHMT1, concurrent demethylation of both K450/K451 led to a remarkable expansion of H3K9-independent chromatin recruitment of EHMT1. Consequently, EHMT1 transcriptionally activated oncogenic programs, promoting prostate cancer development. Overall, our results provide novel molecular insights into the function and regulation of EHMTs, highlighting the potential of targeting EHMTs as a treatment strategy for CRPC.

## Materials and Methods

### Cell Culture, Transfection, and Establishment of Stable Cell Lines

VCaP, LNCaP, CWR-22Rv1, and PC-3 cell lines were obtained from the ATCC. All cell lines were frequently examined for *Mycoplasma* contamination using the MycoAlert Mycoplasma Detection Kit (Lonza) and authenticated using short tandem repeat profiling. VCaP cells were grown in DMEM with 10% FBS. LNCaP, PC-3, and 22RV1 cells were grown in RPMI medium with 10% FBS. For transient transfection of siRNA, cells were transfected with 20 nmol/L of siRNAs (non-target control or siEHMT1/2) for 2 days. siRNA pools were predesigned and obtained from Dharmacon RNAi Technologies (ON-TARGETplus). For constructing the EHMT1-expressing plasmids, we first inserted EHMT1 ORF (GeneCopeia, GC-Y4560-CF) into tetracycline-inducible expression constructs (pLIX_403, Addgene) to generate wild-type (WT) EHMT1, and then used the QuickChange Lightening Site-Directed Mutagenesis Kit (Agilent Technologies) to generate the EHMT1 mutants. These plasmids were then used to assemble lentivirus, which were packaged with pVsVg and pDelta8.9 packaging plasmids (Addgene) in 293T cells. The supernatant was collected after 72 hours and then used to infect LNCaP cells with polybrene (4 μg/mL), followed by puromycin selection (1 μg/mL). All stable cell lines were maintained with tetracycline-free FBS and treated with doxycycline (0.1–0.5 μg/mL for at least 2 days) to induce expression. To generate PC-3-GFP–expressing stable cell lines, we infected PC-3 cells with GIPZ lentiviral particles (purchased from Horizon Discovery).

### Cell Proliferation Assay

Cells were collected and trypsinized to examine the number of cells and viability by using the Muse Count & Viability Kit 200X and following the manufacture's protocol using Guava Muse Cell Analyzer.

### Migration Assay

Transwell migration assays were performed with 24-multiwell Corning FluoroBlok Cell Culture Inserts (Corning, 351152). The same number of cells were seeded in the upper chamber with serum-free medium and the lower chamber was filled with medium containing 10% FBS as a chemoattractant. After 2 days incubation, migrated cells were stained by Corning Calcein AM Fluorescent Dye (Corning, 354217), detected by EVOS autofluorescence microscope, and counted using ImageJ software.

### *In Vitro* Demethylation Assay

Formaldehyde production was measured using the Histone Demethylase Assay kit (Active Motif). Synthetic EHMT1 peptides (441–461aa) with methylated K450 and/or K451(GenScript, with >98% purity) were incubated with recombinant LSD1 (Active Motif) in the demethylation buffer for 1 hour at 37°C, and then detection buffer for 1 hour at 37°C, followed by fluorescence detection.

### Luciferase Reporter Assay

COS7 cells were transfected with a *Firefly* luciferase reporter construct containing approximately 800 bp promoter of MCM7 gene together with a *Renilla* luciferase reporter construct for 48 hours. The activities of *Firefly* luciferase and *Renilla* luciferase were measured using the dual-luciferase reporter assay (Promega), and the results were normalized for *Renilla* activities.

### Immunoprecipitation and Immunoblotting

For the methyl-lysine pulldown assay, GST-3xMBT protein (the GST-3xMBT plasmid is a gift from Dr. Or Gozani) was expressed in *E. coli* DH5α cells and purified by glutathione Sepharose 4B chromatography following the manufacturer's protocol (Amersham Biosciences). Nuclear extracts from VCaP cells treated with or without 1 mmol/L pargyline were used for the pulldown assay with GST-3xMBT protein and the immunopurified proteins were separated by SDS-PAGE gel, stained with Coomassie blue, and then subjected to mass spectrometry analysis. For other immunoprecipitation (IP) assays, cells were lysed in Triton Lysis buffer and treated with protein inhibitor cocktails (Thermo Fisher Scientific), followed by a brief sonication, and then lysates were immunoprecipitated with anti-V5-conjugated beads (Sigma, A7345) or anti-FLAG-conjugated beads (Sigma, A2220). For immunoblotting, proteins were detected with primary antibodies, including anti-V5 (Abcam, ab9116), anti-EHMT1 (Abcam, ab41969), anti-EHMT2 (Novus, NBP2-13948), anti-FLAG (Sigma, F3040), anti-LSD1 (Abcam, ab17721), anti-CoREST (Abcam, ab183711), anti-HDAC1 (Abcam, ab7028), anti-HDAC2 (Abcam, ab7029), anti-REST (Millipore, CS200555), anti-E2F1 (Cell Signaling Technology, 3742), anti-GAPDH (Abcam, ab8245), anti-H3K9me1(Abcam, 9045), anti-H3K9me2 (Cell Signaling, 9753S), and anti-Tubulin (Abcam, ab6046).

### Chromatin Immunoprecipitation Sequencing

For the preparation of chromatin immunoprecipitation sequencing (ChIP-seq), cells were fixed with 1% formaldehyde and then lysed by the ChIP lysis buffer. Chromatin was then sheared into approximately 200–300 bp fragments (∼500–800 bp for ChIP-qPCR) using Bioruptor Sonicator (Diagenode). IP was carried out using antibodies including anti-V5 (Invitrogen, 46-1157), anti-H3K9me2 (Abcam, ab1220), and anti-EHMT1 (Abcam, ab63161). DNA libraries were constructed using the SMARTer ThruPLEX DNA-Seq Prep Kit (Takara Bio). Next-generation sequencing (51 nt, single-end) was performed using the Illumina NextSeq 2000. ChIP-seq reads were mapped to the hg19 human reference genome and peak calling was performed using MACS2.

### qRT-PCR and RNA Sequencing

RNA was extracted using the RNeasy Kit (Qiagen). qRT-PCR was performed using Fast 1-step Mix (Thermo Fisher Scientific). All Taqman primers and probes were predesigned and obtained from Thermo Fisher Scientific. All qRT-PCR data were normalized with internal control, GAPDH. RNA sequencing (RNA-seq) libraries were constructed using TruSeq the Strnd Total RNA LT (Illumina), and sequencing was performed using Illumina NextSeq 2000 (51 nt, single-end). Reads were mapped to the hg19 human reference genome, and gene expression levels were quantified using the R package limma.

### Zebrafish Embryo Metastasis Assay

Zebrafish embryos were generated from AB and TUE WT lines via natural spawning. All experiments were performed in 3 days after fertilization embryos, following a protocol approved by the University of Massachusetts Boston Institutional Animal Care and Use Committee (IACUC). The embryos were dechorionated and anesthetized with 0.04 mg/mL tricaine. Approximately 100 GFP-expressing cells were microinjected into the perivitelline space of each embryo. After injection, the embryos were washed and maintained in 96-well plates at 28°C. Within 1 hour after injection, the embryos were imaged.

### Xenograft Study

All mouse xenograft studies were conducted in compliance with institutional and U.S. national guidelines with approval from the University of Massachusetts Boston IACUC. To establish the xenograft tumors, prostate cancer cells mixed with Matrigel (BD Biosciences) were subcutaneously injected (2 × 10^6^ cells per injection) into the flanks of castrated male SCID mice (∼4–6 weeks, Taconic). Once tumors were established, mice bearing tumors were treated with EHMT1/2 inhibitors via daily oral gavage for 5 days per week. Tumor volume was measured using caliper and calculated using the formula (*L* × *W*^2^/2, *L*: length, *W*: width).

### Statistical Analysis

Data presented in bar graphs represent the mean ± SD of at least three biological repeats. Statistical analysis was performed using an unpaired two-tailed Student *t* test comparing treatment versus vehicle or as otherwise indicated. A *P* value of less than 0.05 was considered to be statistically significant (*, <0.05; **, <0.01; ***, <0.001; ****, <0.0001). Immunoblotting results are representative of at least three experiments. Boxplots of signature scores and gene expression were compared using the Wilcoxon test for comparison between two groups of samples. The difference in tumor growth was determined using two-way ANOVA. These tests were parametric and based on the assumption of normal distribution and equal variance across all experimental groups. The zebrafish metastasis data were analyzed using Fisher exact test or *χ*^2^ test depending on sample size. All statistical analyses and visualization were performed with R (version 3.6.0) unless otherwise specified.

### Data Availability Statement

The ChIP-seq and RNA-seq data have been deposited in Gene Expression Omnibus with the accession number GSE201771, allowing access to the data.

## Results

### EHMT1 and EHMT2 Promote Prostate Cancer Development

**FIGURE 1:**
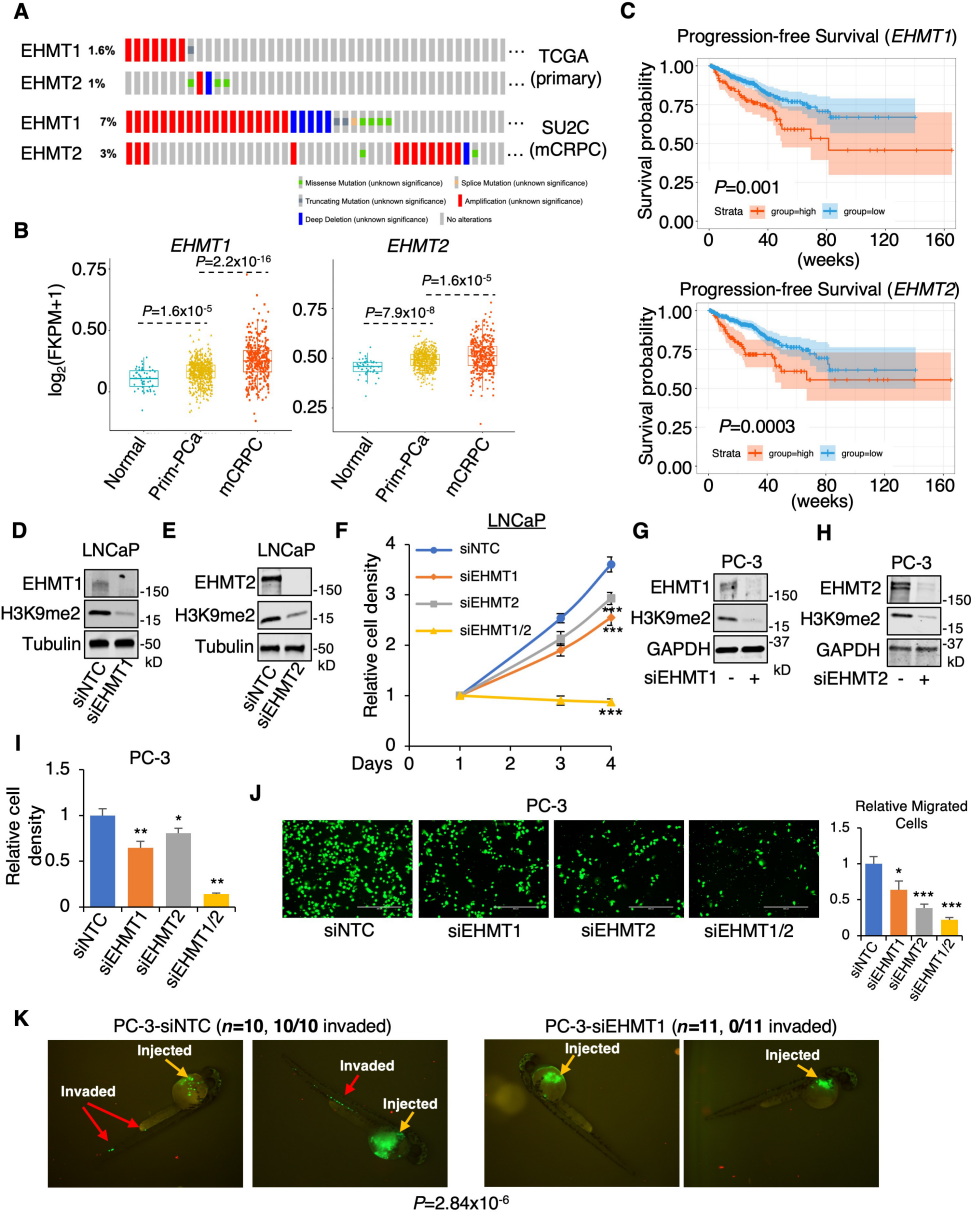
EHMT1 and EHMT2 promote prostate cancer (PCa) development. **A,***EHMT1* and *EHMT2* gene alterations in two prostate cancer cohorts (TCGA primary prostate cancer and SU2C mCRPC). **B,** Box plots depicting the expression levels of *EHMT1* or *EHMT2* in normal and primary prostate cancer samples (TCGA) and mCRPC samples (SU2C). **C,** Kaplan–Meier curve illustrating the overall survival in prostate cancer tumors with higher *EHMT1* or *EHMT2* expression (red, the top 25%) compared with those with lower expression (blue, the bottom 75%). Immunoblotting analysis of indicated proteins in LNCaP cells transfected with siRNA pools against EHMT1 (**D**) or EHMT2 (**E**) for 2 days. **F,** Proliferation assay conducted on LNCaP cells transfected with siEHMT1, siEHMT2, or the combination for 0–4 days. Immunoblotting analysis of indicated proteins in PC-3 cells transfected with siEHMT1 (**G**) or siEHMT2 (**H**) for 2 days. Proliferation assay (**I**) and transwell migration assay (**J**) performed on PC-3 cells transfected with siEHMT1, siEHMT2, or the combination for 4 days (proliferation) and 3 days (migration). **K,** PC-3 cells (stably expressing GFP) transfected with siNTC versus siEHMT1 (for 3 days) were injected into zebrafish embryos (*N* = 10), and tumor cell intravasation was observed using a fluorescence microscope. The number of embryos showing invasion signals was counted. TCGA, The Cancer Genome Atlas.

Analysis of public prostate cancer datasets (12, 24, 25) revealed that the *EHMT1* gene is altered in 1.6% of primary prostate cancer samples, and the alteration rate is increased to 7% in CRPC, with the majority being gene amplification (Fig. 1A). Consistently, *EHMT1* expression is significantly increased in CRPC compared with primary prostate cancer and normal prostate samples (Fig. 1B), suggesting that *EHMT1* may function to promote the progression of CRPC. The genetic alteration and expression of *EHMT2* are also increased in CRPC samples, with the alteration rate rising from 1% to 3%. Furthermore, we investigated whether *EHMT1/2* expression is associated with the aggressiveness of prostate cancer tumors. As shown in Fig. 1C, higher expression of these genes was significantly correlated with poorer clinical outcomes in patients, indicating their potential oncogenic functions in prostate cancer.

To determine the role of EHMT1 and EHMT2 in prostate cancer progression, we examined their protein levels in adenocarcinoma prostate cancer lines (LNCaP, 22Rv1) and stem cell or neuroendocrine (NE)-like prostate cancer lines (PC-3, NCI-H660). EHMT1 protein levels were comparable among these cell lines, while EHMT2 protein was more highly expressed in NCI-H660 cells (Supplementary Fig. S1A). Proliferation assays were performed in LNCaP and PC-3 cell lines after silencing EHMT1 or EHMT2. As shown in Fig. 1D–I and Supplementary Fig. S1B and S1C, the growth of these prostate cancer cells was reduced upon knockdown of either EHMT1 or EHMT2. Importantly, simultaneous silencing of both EHMT1 and EHMT2 resulted in a more pronounced reduction in cell proliferation in both models. Silencing EHMT1 or EHMT2 in PC-3 cells also significantly decreased cancer cell migration (Fig. 1J). Consistent with the effect on cell growth, a synergistic effect was also observed when both EHMT1 and EHMT are silenced simultaneously. Similar effects on cell migration upon EHMT1 or EHMT2 silencing were also observed in LNCaP and 22Rv1 cells (Supplementary Fig. S1D--S1G). In addition, we investigated whether EHMT1 silencing could affect early steps of the metastasis cascade *in vivo* by injecting PC-3 cells into immune-deficient zebrafish embryos (Fig. 1K). While control cells quickly invaded the blood vessel within an hour postinjection (10 out of 10 embryos invaded), EHMT1-silenced cells remained within the perivitelline space of each embryo (0 out of 11 embryos invaded), indicating a critical role of EHMT1 in the dissemination of prostate cancer cell.

### EHMT1 and EHMT2 Transcriptionally Activate Oncogenic Programs

**FIGURE 2:**
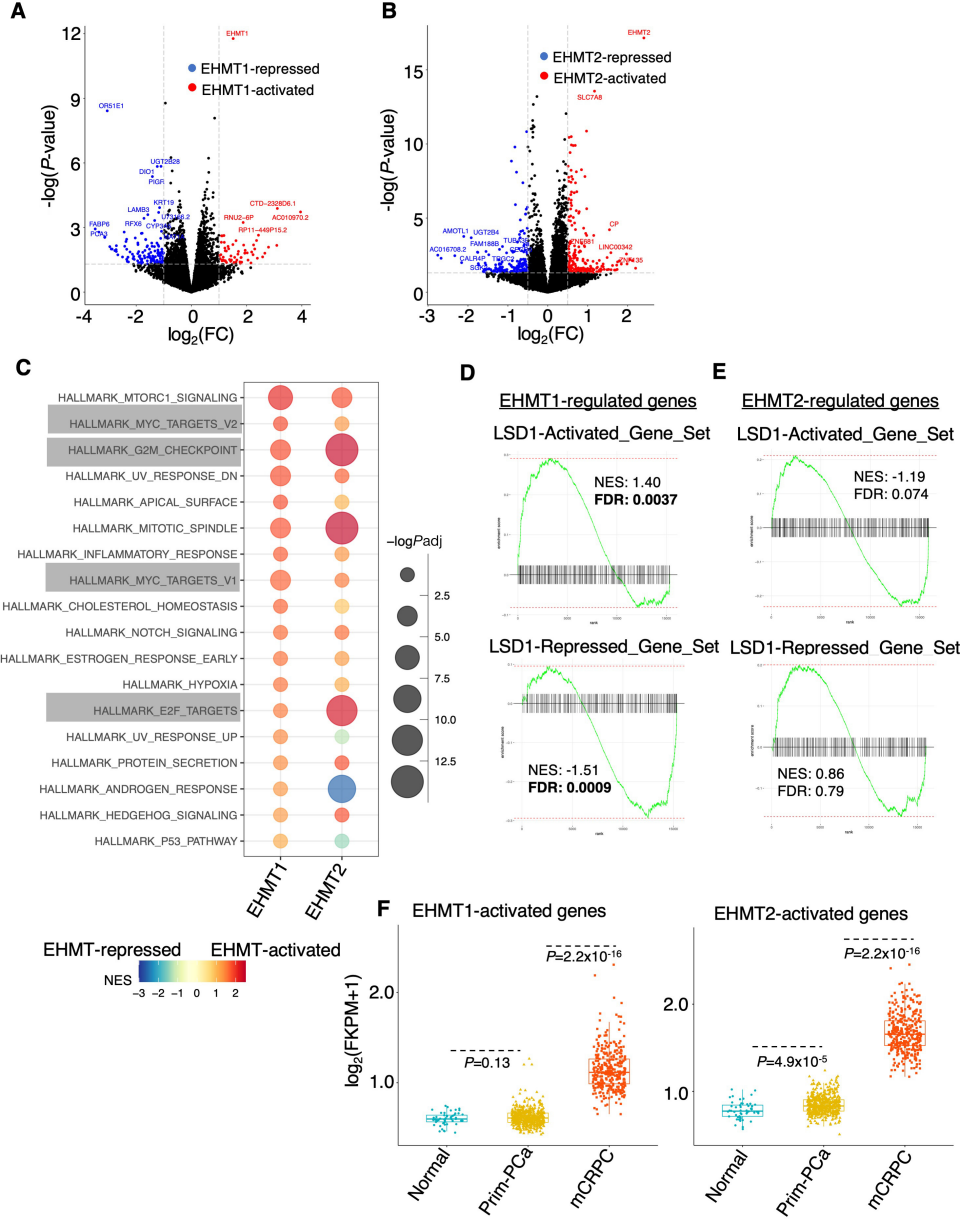
EHMT1 and EHMT2 transcriptionally activate oncogenic programs. **A** and **B,** RNA-seq analyses were conducted in LNCaP cells transfected with siEHMT1, siEHMT2, or siNTC for 2 days. Volcano plots show genes regulated by EHMT1 (**A**) or EHMT2 (**B**). **C,**GSEA of EHMT1 or EHMT2-upregulated genes (siEHMT1 or siEHMT2-downregulated) for hallmark gene set (cutoff: *P_adj_* < 0.05). **D,** GSEA of EHMT1-regulated genes to assess the enrichment of LSD1 function, based on previously determined LSD1 target gene sets (32) and using RNA-seq from GSE52201. **E,** GSEA of EHMT2-regulated genes to evaluate the enrichment of LSD1 function. **F,** Box plots presenting the expression levels of EHMT1-activated or EHMT2-activated genes (cutoff: 2-fold, *P* < 0.05) in normal and primary prostate cancer samples (TCGA) as well as mCRPC samples (SU2C). TCGA, The Cancer Genome Atlas.

To determine the transcription output of EHMTs, we specifically silenced EHMT1 or EHMT2 expression in LNCaP cells and performed RNA-seq analyses to identify differentially regulated genes (Fig. 2A and B). Subsequently, we conducted gene set enrichment analysis (GSEA) using hallmark gene sets to characterize the function of their target genes. Importantly, both EHMT1- and EHMT2-upregulated genes were enriched for multiple oncogenic pathways, including E2F and MYC signaling (Fig. 2C). E2F transcription factors primarily regulate cell-cycle progression, proliferation (26), as well as angiogenesis and metastasis (27). MYC also plays a role in regulating cancer cell motility and metastasis (27, 28) and is a critical oncogenic driver of prostate cancer (29).

Considering that EHMTs are part of the REST complex responsible for repressing neuronal genes, we further investigated whether EHMT1/2-regulated genes were associated with NE transformation in prostate cancer. Surprisingly, EHMT1-regulated genes did not show significant enrichment for previously defined NE gene signatures (30), while EHMT2-activated genes were enriched for an NE-upregulated gene signature, and EHMT2-repressed genes were enriched for an NE-downregulated gene signature (Supplementary Fig. S2A and S2B). In addition, we examined multiple NE markers and found that only EHMT2 could activate the well-characterized NE marker, *SYP*, while both EHMT1 and EHMT2 activated *ENO2* expression (Supplementary Fig. S2C). These findings suggest that EHMT2 may have an uncharacterized role in promoting, but not repressing, NE progression in prostate cancer cells, whereas the involvement of EHMT1 in NE regulation remains uncertain. Because EHMTs can interact with LSD1, and LSD1 can function as a transcription activator inducing oncogenic reprogramming in prostate cancer cells (8, 31), we investigated whether EHMTs and LSD1 shared target genes. To test this, we developed two gene sets for LSD1-activated and -repressed targets based on previous RNA-seq analysis in LNCaP cells (32), and performed GSEA using these defined gene sets. As shown in Fig. 2D, EHMT1-upregulated genes were enriched for LSD1-activated targets, whereas EHMT1-downregulated genes were enriched for LSD1-repressed targets. In contrast, EHMT2-regulated genes did not show significant enrichment for the LSD1 pathway (Fig. 2E). These data suggest that EHMT1 may be more directly associated with LSD1 and may function to activate gene transcription.

Furthermore, we examined the levels of this novel activation activity of EHMT1/2 in prostate cancer patient samples. As shown in Fig. 2F, the expression levels of EHMT1- or EHMT2-activated genes were significantly increased in CRPC compared with primary prostate cancer and normal prostate samples. Overall, these results provide evidence for a possible noncanonical transcriptional activation function of EHMT1 and EHMT2, which can induce oncogenic transcription programs mediated by E2F and MYC.

### A Specific EHMT1/2 Dual Inhibitor Suppresses Prostate Cancer Growth and Metastasis *In Vitro* and *In Vivo*

**FIGURE 3:**
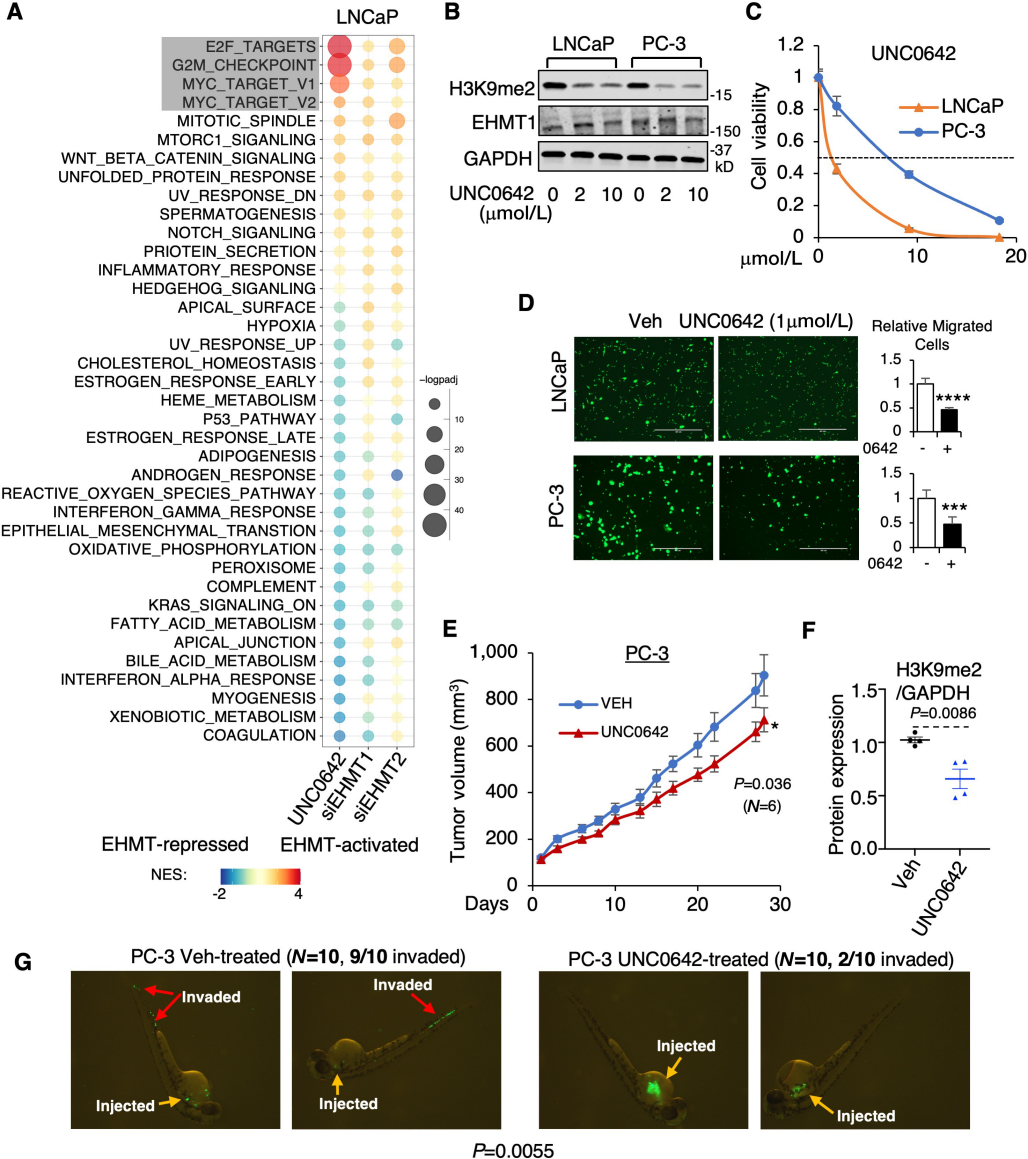
A specific EHMT1/2 dual inhibitor suppresses prostate cancer growth and metastasis *in vitro* and *in vivo.***A,** GSEA conducted on differentially regulated genes by UNC0642 (2 μmol/L, 2 days), siEHMT1, or siEHMT2 (based on RNA-seq analyses). **B,** Immunoblotting analysis of indicated proteins in LNCaP or PC-3 cells treated with UNC0642 (0, 2, 10 μmol/L, 2 days). **C,** Proliferation assay performed on LNCaP or PC-3 cells treated with UNC0642 (0–20 μmol/L, 3 days). **D,** Transwell migration assay conducted on LNCaP or PC-3 cells treated with UNC0642 (1 μmol/L, 2 days). **E,** PC-3 cells were subcutaneously injected into male SCID mice. Once the tumor was established, mice (*N* = 6) were treated with UNC0642 (10 mg/kg) via oral gavage, and tumor volume was measured by caliper. **F,** Normalized H3K9me2 expression was calculated (using ImageJ for quantification) based on immunoblotting of H3K9me2 and GAPDH in tumor samples. **G,** PC-3 cells (stably expressing GFP) pretreated with UNC0642 (2 μmol/L, 2 days) were injected into zebrafish embryos (*N* = 10 for the control and treatment groups), which were subsequently monitored for the fluorescence signal in their circulation system.

Next, we determined whether inhibition of EHMT1/2 enzyme activity by small molecular inhibitors can repress prostate cancer tumor growth and metastasis *in vitro* and *in vivo*. We first tested UNC0642, a potent and catalytic dual inhibitor of EHMT1/2 (33), in various prostate cancer models. The RNA-seq analysis revealed that this inhibitor had a stronger effect on targeting E2F and MYC pathways in LNCaP cells compared with the individual knockdown of EHMT1 or EHMT2 (Fig. 3A). Consistent with the effect on these oncogenic pathways, UNC0642 can significantly decrease *in vitro* cell proliferation and migration in LNCaP and PC-3 cells (Fig. 3B–D). We then generated CRPC xenograft tumors using the PC-3 model and examined the effect of UNC0642 treatment on tumor growth. As shown in Fig. 3E and F, UNC0642 reduced the tumor growth *in vivo*, and the treatment also resulted in the global repression of H3K9me2. Moreover, we also examined this treatment in the zebrafish embryo metastasis model. As shown in Fig. 3G, UNC0642 markedly decreased the metastatic capability of PC-3 cells *in vivo*, indicating that targeting EHMT1/2 may prevent prostate cancer progression and metastasis.

In addition, we also tested two other dual inhibitors, BIX01294 and UNC0638 (34, 35). Both inhibitors potently suppressed prostate cancer cell growth and migration *in vitro* (Supplementary Fig. S3A–S3H). However, in contrast to UNC0642, these two inhibitors appear to be not optimized for use *in vivo* because we cannot detect any changes in H3K9me2 levels in CRPC xenograft models (Supplementary Fig. S4A–S4F). Indeed, they were previously reported to have poor pharmacokinetic properties (23). All three inhibitors were well tolerated by SCID mice (Supplementary Fig. S4G). Overall, these studies suggest a strong therapeutic potential for using dual inhibitors of EHMT1 and EHMT2 for treating prostate cancer.

### Lysine 450 and 451 of EHMT1 are Methylated

**FIGURE 4:**
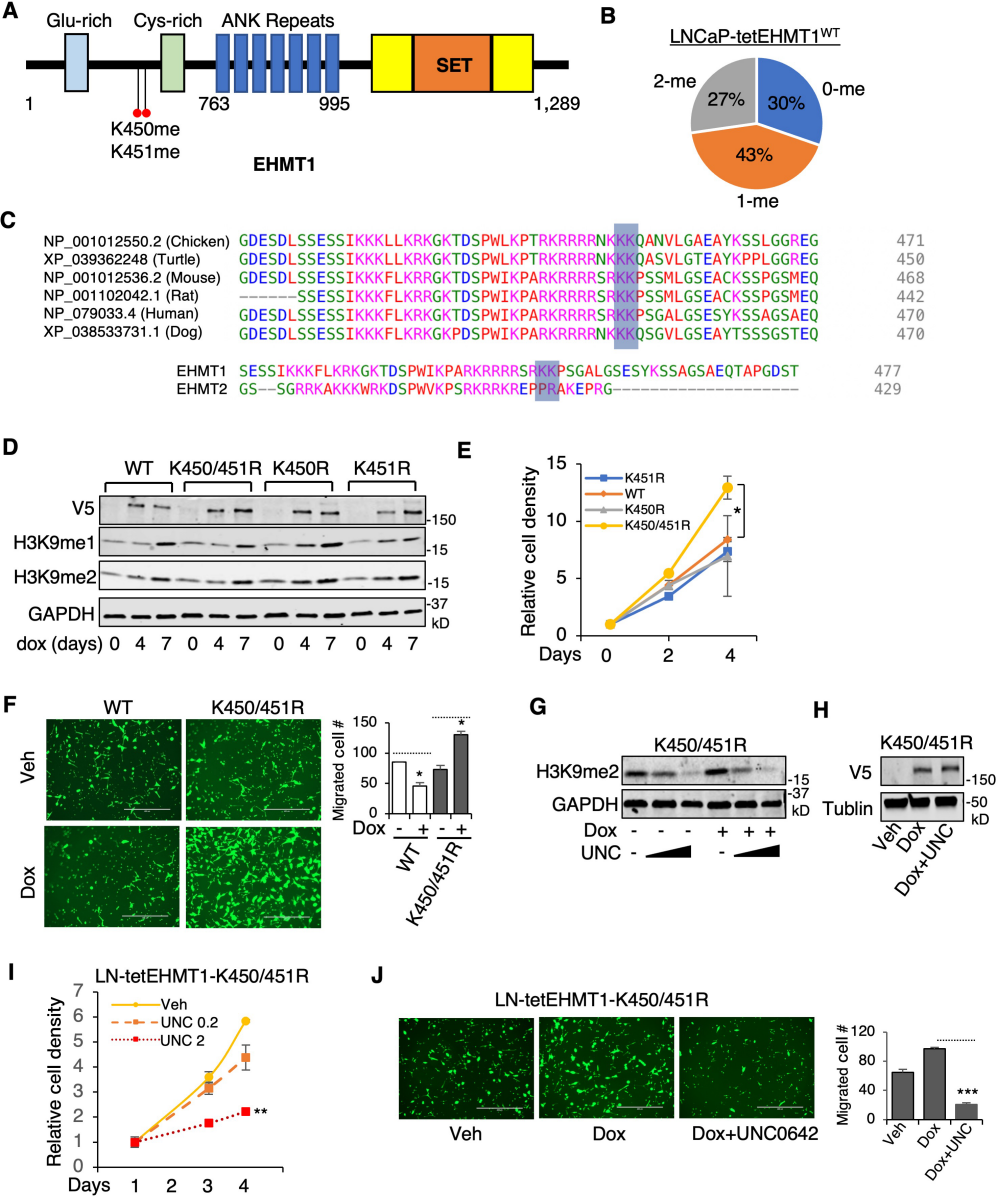
Concurrent demethylation of K450 and K451 enhances EHMT1 oncogenic activity. **A,** Mass spectrometry analysis on immunoprecipitated V5-EHMT1 in LNCaP cells stably overexpressing V5-tagged doxycycline-regulated EHMT1 (treated with doxycycline for 2 days) revealed methylations at K450 and K451 sites. **B,** The pie chart illustrating the percentage of detected unmethylated peptides (0-me), K450 or K451 methylated peptides (1-me), and K450/K451 dual-methylated peptides (2-me). **C,** Amino acid alignment of EHMT1 K450/451 surrounding amino acid sequence across different species and with human EHMT2 protein. **D,** Immunoblotting analysis of V5 in LNCaP cells stably overexpressing V5-tagged doxycycline-regulated EHMT1-WT, K450R, K451R, and K450/451R treated with doxycycline for 0–7 days. **E,** Proliferation assay performed on LNCaP stable cells treated with doxycycline for 1–5 days at 0.1 μg/mL. **F,** Transwell migration assay conducted on LNCaP stable cells expressing EHMT1 WT versus K450/451R mutant treated with or without doxycycline for 3 days. **G,** Immunoblotting analysis of H3K9me2 in EHMT1-K450/451R cells treated with doxycycline (for 3 days) and/or UNC0642 (0, 0.2, 2 μmol/L, for 2 days). **H,** Immunoblotting analysis of V5 in EHMT1-K450/451R cells treated with doxycycline (for 3 days) and/or UNC0642 (2 μmol/L, for 2 days). **I,** Proliferation assay performed on EHMT1-K450/451R cells treated with doxycycline and/or UNC0642 (0.2 or 2 μmol/L, for 1–4 days). **J,** Transwell migration assay conducted on EHMT1-K450/451R cells treated with doxycycline (for 3 days) and/or UNC0642 (2 μmol/L, for 2 days).

To gain further insights into the posttranslational regulation of EHMT1, we generated a stable LNCaP cell line expressing V5-tagged EHMT1 under doxycycline regulation. Mass spectrometry analysis of immunopurified V5-EHMT1 protein (Supplementary Fig. S5A) revealed strong methylation signals on lysine residues 450 and 451, with approximately 70% peptide reads showing either single or double methylation (Fig. 4A and B). Interestingly, this double-lysine site was highly conserved across different species but was not found in the EHMT2 protein (Fig. 4C).

To investigate the functional implications of K450/K451 methylation in prostate cancer cells, we generated three additional LNCaP stable cell lines expressing doxycycline-regulated V5-tagged EHMT1-K450R, K451R, or K450/451R mutants. Short-term doxycycline treatment induced similar expression of mRNA and protein in the mutant cell lines compared to the WT (Supplementary Fig. S5B and S5C). The mutations did not significantly affect the half-life or degradation of the EHMT1 protein (Supplementary Fig. S5D and S5E), suggesting that lysine methylation does not hinder any potential polyubiquitination at these residues (36). Given that the amino acid sequence surrounding K450/451 resembles a classical nuclear localization signal, we also investigated whether the mutations affected the nuclear localization of the EHMT1 protein. Through subcellular fractionation assay and immunofluorescence staining, we found that the mutations did not influence this activity of EHMT1 (Supplementary Fig. S5F and S5G). Furthermore, we examined whether these mutations could alter EHMT1’s methyltransferase activity on H3K9. As shown in Fig. 4D, all three mutants demonstrated a similar increase in cellular levels of H3K9me2 compared with WT EHMT1, indicating that the canonical activity of EHMT1 in methylating H3K9 is not significantly affected by lysine methylation at K450 or K451. Overall, these data indicate that these lysine methylations may regulate previously unknown activities of EHMT1.

### The Dual-lysine Demethylation Promotes Prostate Cancer Development

To further understand the function of K450 and K451 methylation, we examined the growth of the four stable cell lines treated with low-dose doxycycline. As shown in Fig. 4E, while a single mutation did not significantly affect LNCaP cell proliferation, the K450/451R mutant exhibited a significant increase in the proliferation of LNCaP cells, suggesting that unmethylated EHMT1 may confer stronger oncogenic activity. Next, we performed a migration assay to compare the effects of expressing the WT versus K450/451R mutant. As shown in Fig. 4F, overexpression of the K450/451R mutant caused a significant increase in cancer cell migration compared with the WT cell line. To further determine whether these oncogenic functions still require the enzymatic activity of EHMT1, we treated the K450/451R mutant with UNC0642, an inhibitor of EHMT1 enzymatic activity. The inhibitor treatment suppressed the levels of H3K9me2 in prostate cancer cells but did not affect the protein expression of EHMT1 (Fig. 4G and H). Importantly, the enhanced cell proliferation or migration observed in the K450/451R mutant was blocked by EHMT1 inhibition (Fig. 4I and J), suggesting that catalytic activity of EHMT1 is required for the unmethylated form to promote prostate cancer progression.

### The Dual-lysine Demethylation Reprograms EHMT1-mediated Transcription

**FIGURE 5:**
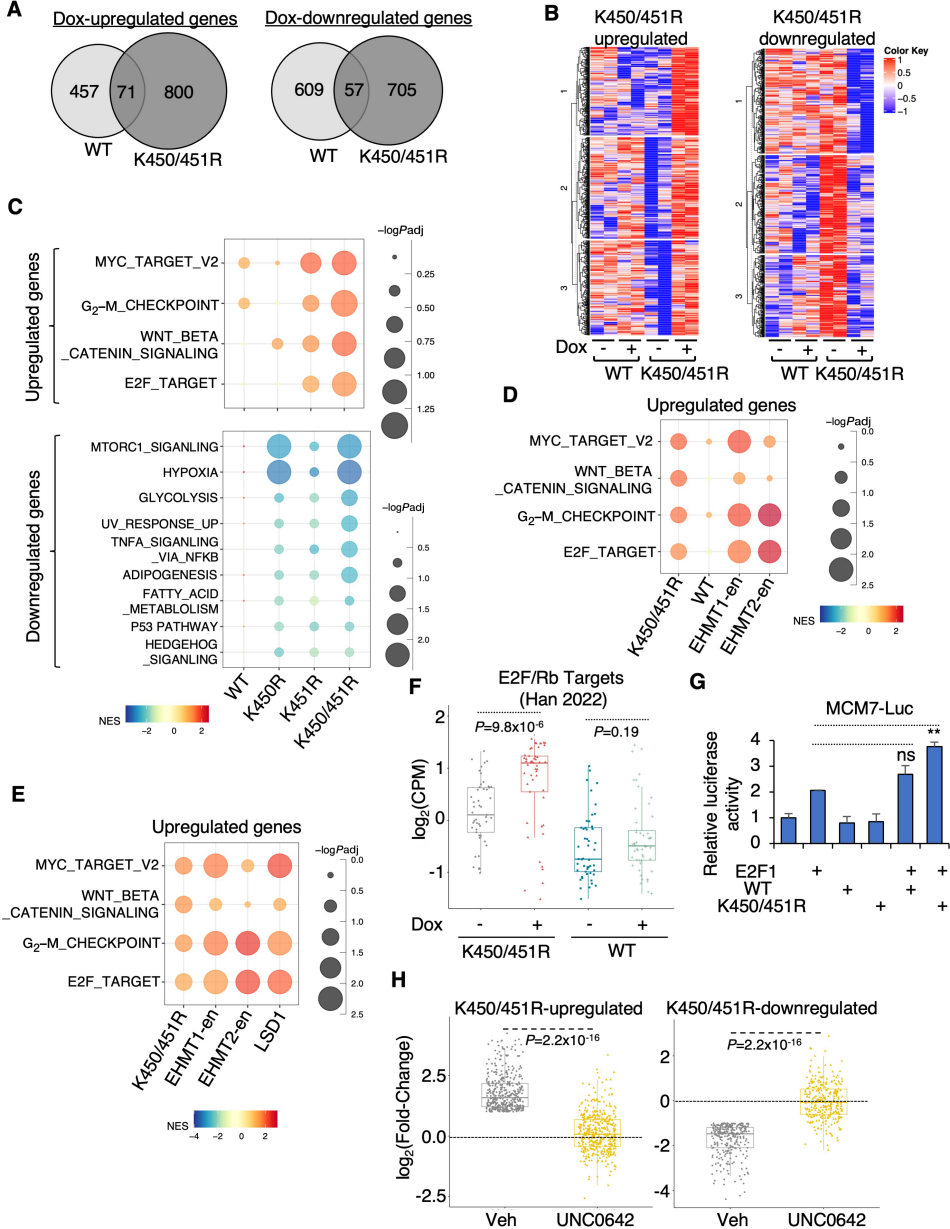
The dual-lysine demethylation reprograms EHMT1-mediated transcription. **A,** Venn diagrams showing EHMT1-WT–regulated genes versus K450/451R-regulated genes (cutoff: 2-fold, *P* < 0.05). **B,** Heat map view presenting K450/451R-regulated genes (cutoff: 2-fold, *P* < 0.05). **C,** GSEA conducted to assess the enrichment of genes regulated by the WT and mutants using hallmark gene sets (cutoff: *P* < 0.05). **D,** GSEA of genes upregulated by the overexpression of WT or K450/451R mutant in the stable lines compared with those downregulated by siEHMT1 or siEHMT2 (cutoff: *P* < 0.05). **E,** GSEA of genes upregulated by the K450/451R mutant compared with genes downregulated by siEHMT1, siEHMT2, or siLSD1 (cutoff: *P* < 0.05). **F,** Box plots depicting the relative expression of previously identified common E2F/Rb targets (49-gene) in EHMT1-K450/451R expressing LNCaP cells. **G,** Luciferase reporter assay measuring MCM7-promoter (E2F1 target) activity in COS7 cells transfected with E2F1 and/or EHMT1-WT or the K450/451R mutant. **H,** Box plots representing the doxycycline-induced change of K450/451R-upregulated or -downregulated genes in EHMT1-K450/451R stable cells (pretreated with doxycycline) treated with or without UNC0642 (2 μmol/L, for 2 days; based on RNA-seq analysis).

To investigate the transcriptional impact of the dual-lysine demethylation of EHMT1, we performed RNA-seq analysis in the stable cell lines expressing WT EHMT1 and the mutants. Comparing the genes upregulated or downregulated by doxycycline in the WT versus mutant-expressing cell lines revealed distinct transcriptional targets (Fig. 5A; Supplementary Fig. S6A–S6C). The difference was further highlighted in a heat map, showing that a significant fraction of genes activated by the K450/451R mutant were not significantly upregulated by the WT EHMT1 (Fig. 5B). This finding was further confirmed with two genes specifically activated by the K450/451R mutant (Supplementary Fig. S6D). We performed GSEA using the EHMT1 stable cell lines and compared the genes upregulated and downregulated by WT, K450R, K451R, or K450/451R. As shown in Fig. 5C and Supplementary Fig. S6E, the K450/451R-upregulated genes were significantly more enriched for several oncogenic pathways including E2F, MYC, and WNT signaling pathways, while the downregulated genes were enriched for MTORC1 and hypoxia pathways. The transcriptional programs activated by the dual-lysine mutant resembled those activated by endogenous EHMT1 or LSD1 (Fig. 5D and E).

To confirm the activation of E2F signaling by the K450/451R mutant, we examined the expression of previously identified prostate cancer–specific E2F/Rb targets (37) and observed broad activation of those genes upon overexpression of the K450/451R mutant (Fig. 5F). In addition, we conducted a luciferase reporter assay using a previously constructed reporter driven by the *MCM7* promoter (38) and found that the dual-lysine mutant further increased E2F1 activity (Fig. 5G). These findings suggest that unmethylated EHMT1 may potentially function as a coactivator of E2F1. To further investigate whether the specific transcription program regulated by the K450/451R mutant still requires the enzymatic activity of EHMT1, we performed RNA-seq analysis in the mutant expressing cells treated with the EHMT1 inhibitor UNC0642. As shown in Fig. 5H, the activation and repression functions of the dual-lysine mutant were markedly impaired when EHMT1’s enzymatic activity was inhibited. These results suggest that unmethylated EHMT1 can specifically activate oncogenic transcription programs, possibly through the methylation of non-H3K9 histone residues or non-histone proteins.

### The Dual-lysine Demethylation Expands EHMT1 Chromatin Occupation

**FIGURE 6:**
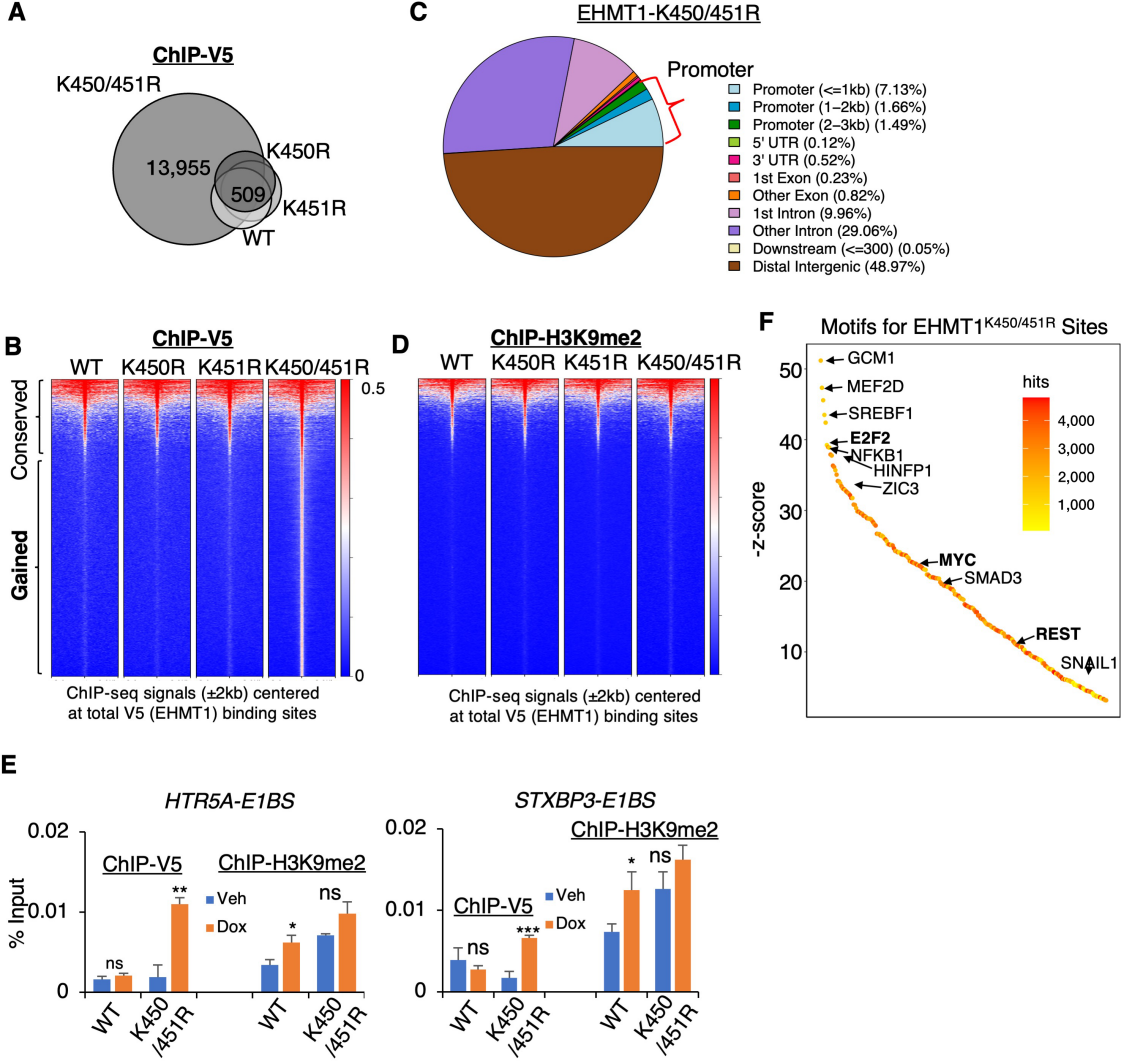
The dual-lysine demethylation dramatically expands EHMT1 chromatin occupation. **A,** ChIP-seq analyses of V5 were conducted on LNCaP stable cells overexpressing EHMT1 WT, K450R, K451R, and K450/451R (doxycycline pretreated for 2 days). The Venn diagram displays the overlapped sites. **B,** Heat map views presenting ChIP-seq signal intensity of V5 at total V5-EHMT1 binding sites. **C,** Chromatin distribution of EHMT1-K450/451R mutant binding sites. **D,** Heat map views presenting ChIP-seq signal intensity of H3K9me2 (obtained from ChIP-H3K9me2) at total V5-EHMT1 binding sites. **E,** ChIP-qPCR conducted for V5 binding (EHMT1 WT or K450/451R mutant) and H3K9me2 levels at two identified mutant binding sites in the stable cell lines. **F,** Motif enrichment analysis performed for ChIP-V5 sites identified in K450/451R mutant expressing cells.

We conducted V5 ChIP-seq analyses for V5-tagged EHMT1 in the four stable cell lines treated with doxycycline to assess the effects of the mutations on the chromatin occupation of EHMT1. The results revealed that the dual-lysine mutation (K450/451R), but not any single-lysine mutation, significantly expanded the chromatin binding sites of EHMT1 (Fig. 6A and B). These newly gained binding sites were highly enriched in the promoter region of genes (Fig. 6C; Supplementary Fig. S7A). In addition, we performed H3K9me2 ChIP-seq analyses in these cell lines and observed that the levels of H3K9me2 at the majority of the gained sites were low and not significantly altered by any of the mutants (Fig. 6D). This suggests that the expanded chromatin recruitment of EHMT1 by the dual-lysine mutant does not lead to further global H3K9 methylation. Several identified EHMT1 sites were validated to support these findings (Fig. 6E).

Because EHMT1 lacks a DNA-binding domain, its interaction with chromatin is likely facilitated by specific transcription factors. To investigate this, we performed motif analysis on the chromatin sites occupied by the K450/451R mutant compared with the single-lysine mutants and WT EHMT1 (Fig. 6F; Supplementary Fig S7B). The most significantly enriched motifs at the K450/451R mutant binding sites included GCM1, MEF2D, SREBF1, E2F2, NFKB1, and HINFP1, as well as MYC. The enrichment of E2F and MYC binding motifs is consistent with the activation of E2F and MYC signaling by the K450/451R mutant. Interestingly, both E2F1 and MYC have been shown to interact with EHMT1’s interacting partner, EHMT2, in previous studies (14, 39). By utilizing published E2F1 and MYC ChIP-seq datasets in LNCaP and LNCaP-derived cells, we identified that a subset of the gained EHMT1 sites might be co-occupied by E2F1 or MYC (Supplementary Fig. S7C and S7D). For instance, *PPP1R14C*, an E2F1 target, which encodes a regulatory inhibitor subunit of protein phosphatase 1 and promotes cell-cycle progression and migration in prostate cancer cells (40), was specifically activated by the K450/451R mutant (Supplementary Fig. S7E–S7G). The induction of *PPP1R14C* was completely abolished when E2F1 was silenced through RNAi. In addition, we detected enrichment of the REST binding motif, consistent with the EHMT1’s role as a component of the REST repressor complex. Overall, these findings indicate that the dual-lysine demethylation of EHMT1 can expand its chromatin recruitment, activate oncogenic transcription programs, and promote prostate cancer cell proliferation and migration.

### EHMT1 can be Partly Demethylated by LSD1

**FIGURE 7:**
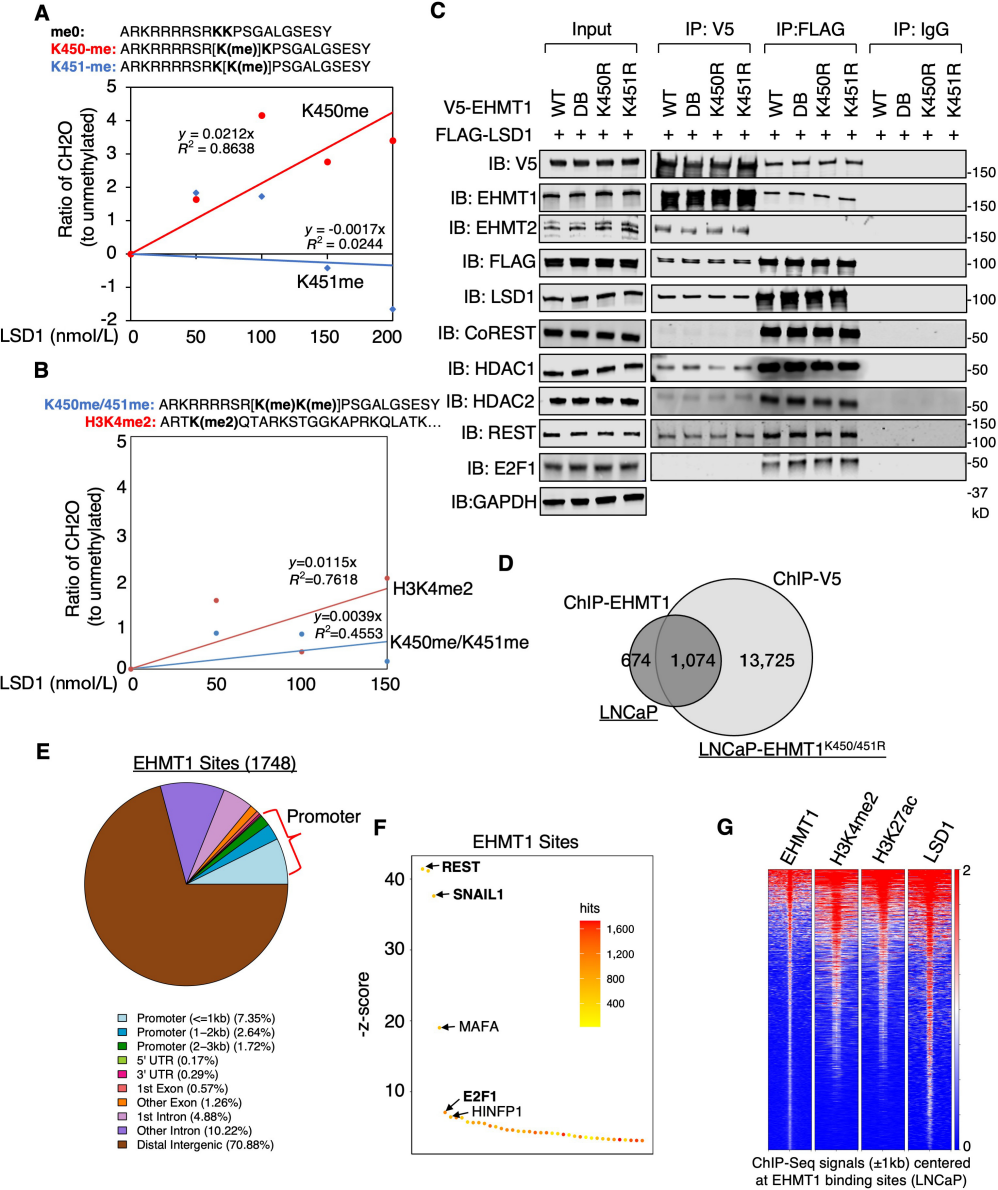
EHMT1 can be partly demethylated by LSD1. *In vitro* demethylation assay measuring formaldehyde production using synthetic K450- and K451-methylated EHMT1 peptides (amino acids 441–461; **A**) or dual-methylated EHMT1 peptide and K4-dimethylated H3 recombinant protein (**B**), as substrates incubated with recombinant LSD1 protein. Results were normalized to the unmethylated EHMT1 peptide. **C,** Immunoblotting analysis of the indicated proteins co-immunoprecipitated with V5 or FLAG in HEK293 cells cotransfected with V5-EHMT1 WT (doxycycline for 3 days) and mutants with FLAG-LSD1. **D,** The Venn diagram illustrating ChIP-EHMT1 peaks in parental LNCaP cells compared with ChIP-V5 peaks in LNCaP cells expressing EHMT1-K450/451R. **E,** Chromatin distribution of EHMT1 binding sites was displayed. **F,** Motif enrichment analysis for EHMT1 binding sites. **G,** Heat map view presenting the ChIP-seq signal intensity of EHMT1, H3K4me2, H3K27ac, and LSD1 centered at EHMT1 binding sites in LNCaP cells.

Given the connection of LSD1 with EHMT1, we hypothesize that EHMT1 could be a substrate of LSD1. To test this, we performed an affinity pulldown assay in VCaP prostate cancer cells using a 3xMBT peptide (Supplementary Fig. S8A), which can strongly interact with methyl-lysine (41). The cells were treated with an irreversible selective LSD1 inhibitor, pargyline (42). Mass spectrometry analysis of the precipitated proteins revealed that EHMT1 and TOP2A exhibited strong interaction with the 3xMBT peptide and showed increased interaction in response to the LSD1 inhibitor (Supplementary Fig. S8B). This analysis also identified novel methylated residues, including K450 and K451 methylations in EHMT1, K219 methylation in SETD2, and K321 methylation in TOP2A (Supplementary Fig. S8C). However, the mass spectrometry analysis could not quantitatively determine which lysine methylation of EHMT1 is increased. To determine the specific lysine residue that could be a substrate of LSD1, an *in vitro* demethylase assay was conducted. The results showed that LSD1 demethylated the K450me-containing peptide but not the K451me-containing peptide (Fig. 7A), indicating that K450me is a potential substrate of LSD1, while K451me may be demethylated by other lysine demethylases. Interestingly, LSD1 activity was also lower for the K450/451 dual-methylated peptide (Fig. 7B), suggesting that the demethylation process may be sequential, with K451 demethylation occurring before LSD1-mediated demethylation of K450. The interactions between EHMT1 and other proteins, including EHMT2, LSD1, HDAC1/2, and REST, were not significantly affected by the mutations at the K450 and/or K451 sites (Fig. 7C). Interestingly, while EHMT2 was reported to be a subunit of an E2F1 complex (14), we did not detect a direct interaction between EHMT1 and E2F1. However, a strong interaction was detected between LSD1 and E2F1, consistent with our previous finding (37).

### EHMT1 Binding is Globally Associated with LSD1

**FIGURE 8:**
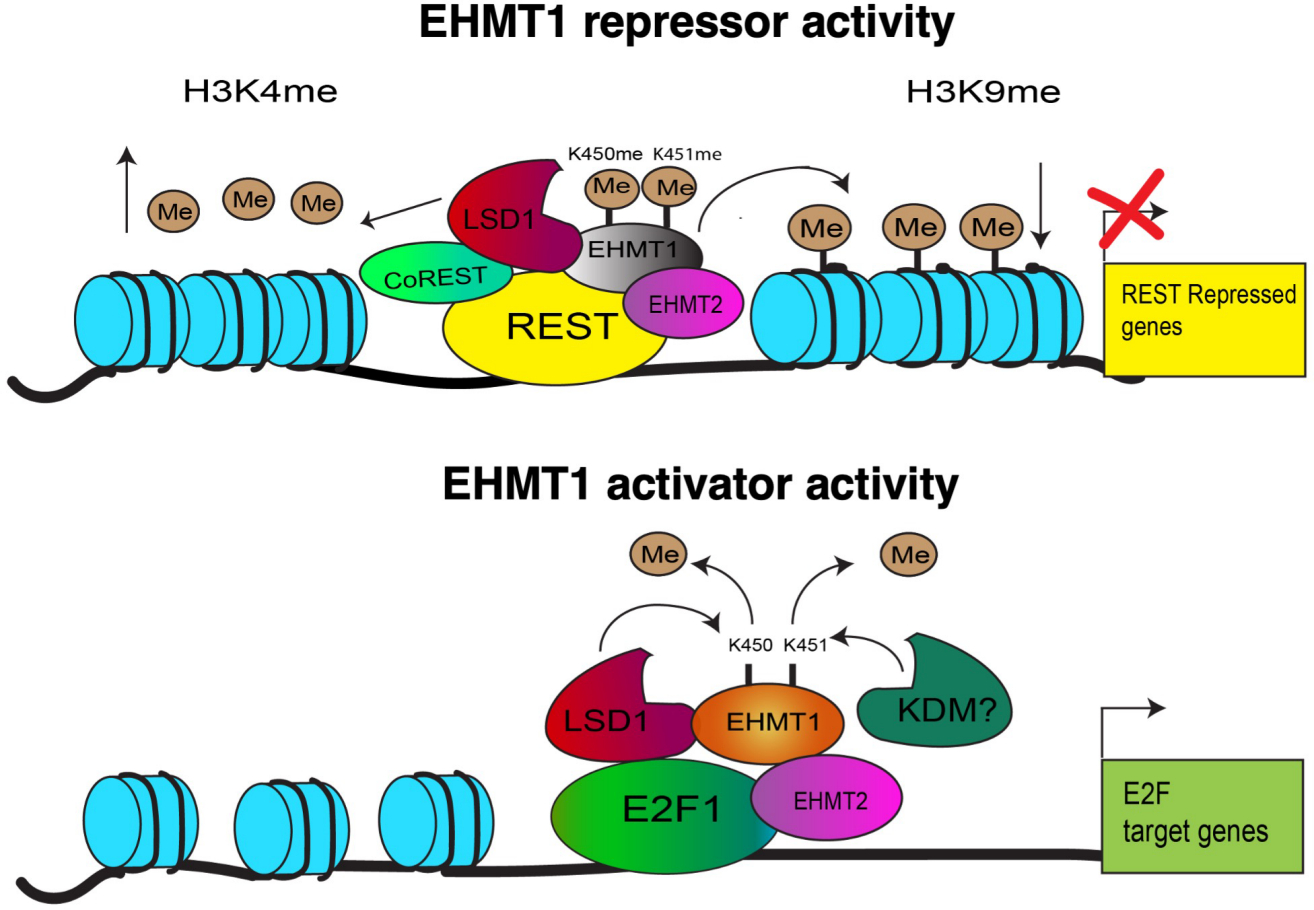
A working model for the transcriptional repressor and activator functions of EHMT1. A graphic model illustrates how lysine demethylation enables the oncogenic activity of EHMT1.

Furthermore, EHMT1 chromatin binding was examined through ChIP-seq analysis in LNCaP cells. Approximately 1,700 binding peaks were identified for the endogenous EHMT1, with approximately 60% and approximately 30% of those sites overlapping with the binding sites of the K450/451R mutant or WT, respectively (Fig. 7D; Supplementary Fig. S8D). Consistently, the endogenous EHMT1 binding was also enriched in gene promoter regions (Fig. 7E). Motif enrichment analysis on these sites showed a significant enrichment of REST, SNAIL1, E2F1, and HINFP1 (Fig. 7F). Importantly, using our previously published ChIP-seq datasets (8, 32), we observed a clear association between EHMT1 binding and enhancer marks (H3K4me2, H3K27ac) as well as LSD1 binding (Fig. 7G). These findings suggest that EHMT1 may be a component of the LSD1 activator complex, and at least a subset of EHMT1 binding is associated with enhancers and transcriptional activation rather than repression. A working model on EHMT1 activity in cancer cells is presented in Fig. 8, based on all the evidence.

## Discussion

This study has identified lysine 450 and 451 methylations as novel posttranslational modifications of EHMT1 and demonstrated the functional importance of dual-lysine demethylation as a molecular switch in reprogramming EHMT1 activity in prostate cancer cells. Interestingly, this H3K9-independent activity of EHMT1 in prostate cancer is similar to the previously reported polycomb-independent activity of EZH2, which is regulated by its phosphorylation at serine 21 (6). These modifications also change the functions of EHMT1 and EZH2 from transcription corepressors to possible coactivators, highlighting the significant role of posttranslational modifications of histone-modifying proteins in mediating epigenetic reprogramming in cancer. Our results indicate that LSD1 may specifically demethylate K450 but not K451, suggesting that this dual-lysine methylation/demethylation process may be precisely regulated by multiple KMTs and KDMs. While LSD1 and EHMTs are known subunits of the REST repressor complex, involved in silencing gene transcription by demethylating and methylating their canonical histone targets (H3K4me1,2 and H3K9; ref. 20), studies from us and other groups have shown that LSD1 can also act as an activator in prostate cancer cells through multiple mechanisms, including the demethylation of non-histone proteins like FOXA1 (8, 31). Interestingly, a previous study reported that EHMT2 can methylate lysine 114 of LSD1, leading to its recognition and interaction with the chromatin-remodeling protein CHD1 to activate gene transcription in prostate cancer cells (19). These modifications between EHMT1/2 and LSD1 may serve as biochemical switches that establish a coactivator network and regulate their coactivator functions, potentially allowing them to functionally collaborate in a transcription activator complex.

One possible activator complex is the AR complex, as K114-methylated LSD1 can activate the classic AR target, *TMPRSS2* (19). However, data from us and others suggest that EHMT1/LSD1 can function as activators in AR-negative prostate cancer cell lines, indicating their potential involvement in an AR-independent complex (7, 37). Another possible transcription activator complex is the E2F complex, as E2F family members were highly ranked in the motif enrichment analysis of EHMT1 binding sites, and the transcriptional programs of EHMT1/2 and LSD1 were significantly enriched for E2F signaling pathways. We also observed that the dual-lysine mutant of EHMT1 specifically enhanced the E2F activity on the MCM7-promoter reporter and an E2F target, *PPP1R14C*. Although we did not detect a direct interaction between EHMT1 and E2F1 in our experiments, a previous study from us revealed the important role of LSD1 in stabilizing E2F1 chromatin binding in prostate cancer cells, and a strong interaction between LSD1 and E2F1 was observed (ref. 37; also see Fig. 7C). Therefore, EHMT1 may indirectly interact with E2Fs through LSD1 or EHMT2. In addition, the strong activity of EHMT1 in promoting prostate cancer metastasis suggests its potential involvement in regulating metastasis-related transcription factors, such as MYC. However, in these possible activator complexes, EHMT1 may function to methylate non-H3K9 substrates. Further studies are needed to fully understand the molecular basis of how K450/451 methylation of EHMT1 affects the potential E2F-LSD1-EHMT1/2 complex (or other activator complexes).

Notably, the amino acid sequence encompassing the K450/K451 region of EHMT1 bears a resemblance to a nuclear localization signal, suggesting a potential role of methylation modifications in regulating EHMT1’s subcellular localization. However, as indicated in Supplementary Fig. S5G, both WT and K450/451R mutant EHMT1 displayed clear nuclear staining, indicating that this mutation does not impact nuclear localization. Interestingly, EHMT1 exhibited puncta formation within the nucleus, a characteristic feature of nuclear condensates typically composed of coactivator complexes (43, 44). Strikingly, the K450/451R mutant showed even more pronounced puncta formation, implying an increased involvement in transcriptional activation functions. Given our recent findings of nuclear condensates between LSD1 and BRD4 driving super-enhancer activation in prostate cancer cells (31), it is plausible that EHMT1 may also participate in these complexes. We are currently conducting further investigations in our laboratory to explore this possibility.

One limitation of this mechanistic study is that we relied solely on the lysine to arginine mutations at K450/451 sites, which preserve the amino acid characteristics of the domain. However, we cannot entirely rule out the possibility that these mutations may create an artificial gain-of-function effect. To address this concern, a further confirmation using lysine to alanine mutations may be pursued, even though alanine substitutions might disrupt domain characteristics. In addition, it is conceivable that this site could undergo other modifications, such as ubiquitination and acetylation, although our mass spectrometry analyses did not detect such modifications. Nonetheless, to fully address these caveats, more comprehensive investigations may be required in the future.

LSD1 inhibitors have been tested in clinical trials for leukemia, small cell lung cancer, and other solid tumors, with them entering phase II trials (e.g., ORY1001 and INCB059872; ref. 45). A previous study also demonstrated the efficacy of a structurally distinct LSD1 inhibitor (SP2509) in treating PC-3–derived xenograft, a stem cell-like CRPC model (7). In contrast, EHMT1/2 inhibitors are still underdeveloped, and none of these inhibitors have been tested in clinical trials for cancer. Our data suggest that UNC0642 may exhibit antitumor activities *in vivo* by moderately repressing CRPC tumor growth and more potently on metastasis. In future studies, EHMT1/2 inhibitors could be combined with LSD1 inhibitors to more effectively repress the oncogenic pathways in CRPC. One potential concern of this treatment strategy would be the possibility of lineage reprogramming induced by EHMT1/2 inhibitors. However, our data reveal that targeting EHMT1/2 can decrease the expression of genes mediating NE progression. Therefore, it is unlikely that the treatment could cause CRPC to transition to NE prostate cancer. Nonetheless, the effects of long-term treatment with these inhibitors would need to be assessed in future research. In summary, our study demonstrates the role of a novel EHMT1 modification as a biochemical switch in mediating its molecular activity and tumor-promoting function in prostate cancer. It also suggests a novel therapeutic strategy in CRPC by directly targeting EHMTs or concurrently targeting EHMTs and other epigenetic factors, such as LSD1.

## Supplementary Material

Figure S1 Figure S1 shows that Silencing EHMT1 or EHMT2 decreases PCa cell proliferation and migration.

Figure S2 Figure S2 shows that EHMT2 activates genes mediating neuroendocrine transformation in PCa.

Figure S3 Figure S3 shows that EHMT1/2 inhibitors BIX01294 and UNC0638 decrease LNCaP and PC-3 cell proliferation and migration.

Figure S4 Figure S4 shows that EHMT1/2 inhibitors BIX01294 and UNC0638 cannot suppress CRPC xenograft tumors in vivo.

Figure S5 Figure S5 shows that K450/K451 methylations do not affect EHMT1 protein stability or nuclear localization.

Figure S6 Figure S6 shows that Transcription profiling for WT and mutant EHMT1-overexpressing cells.

Figure S7 Figure S7 shows that K450/451R mutant-induced expression of PP1R14C can be diminished by silencing E2F1.

Figure S8 Figure S8 shows that Mass-spectrometry analysis on 3xMBT-purified proteins in PCa cells treated with LSD1 inhibitor.
