# Supplementary material for: Demethylation of EHMT1/GLP Protein Reprograms Its Transcriptional Activity and Promotes Prostate Cancer Progression
Source: Cancer Res Commun. 2023 Aug 31;3(8):1716–30. doi: 10.1158/2767-9764.CRC-23-0208 (PMC10470473; doi:10.1158/2767-9764.CRC-23-0208)
Supplement: Figure S1 — shows that Silencing EHMT1 or EHMT2 decreases PCa cell proliferation and migration. [file crc-23-0208-s01.pdf]

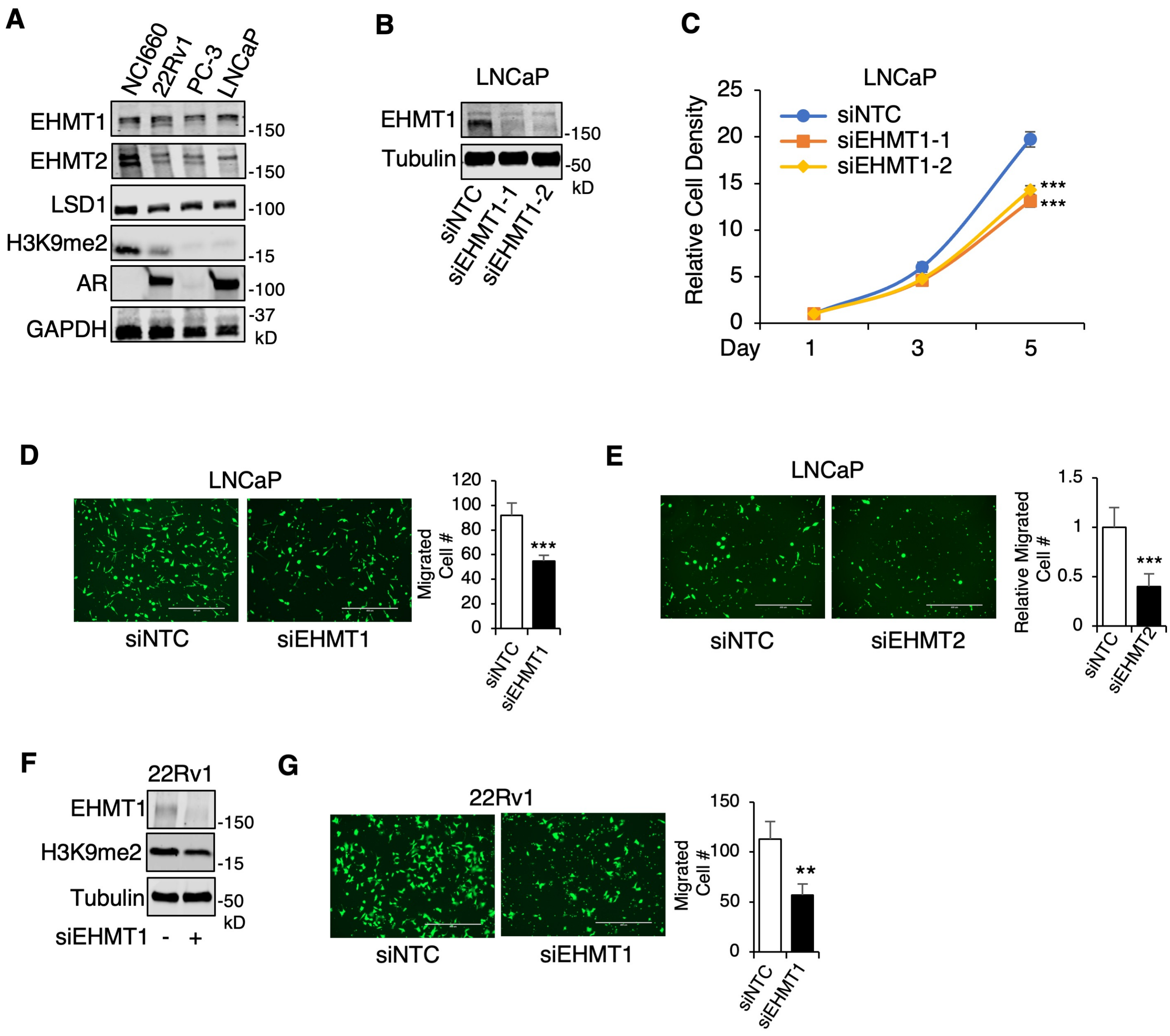

Supplementary Figure S1. Silencing EHMT1 or EHMT2 decreases PCa cell proliferation and migration

(**A**) Immunoblotting for the indicated proteins in four PCa cell lines. (**B**, **C**) Immunoblotting for EHMT1 (B) and proliferation assay (C) in LNCaP cells transfected with two individual siRNAs against EHMT1 (3d for immunoblotting, 1-5d for proliferation assay). (**D**, **E**) Transwell migration assay in LNCaP cells transfected with siRNA pools against EHMT1 (D) or EHMT2 (E) for 3d. (**F**, **G**) Immunoblotting for EHMT1 (F) and transwell migration assay (G) for CWR-22RV1 cells transfected with siEHMT1 or siNTC for 3d.
