# Supplementary material for: Demethylation of EHMT1/GLP Protein Reprograms Its Transcriptional Activity and Promotes Prostate Cancer Progression
Source: Cancer Res Commun. 2023 Aug 31;3(8):1716–30. doi: 10.1158/2767-9764.CRC-23-0208 (PMC10470473; doi:10.1158/2767-9764.CRC-23-0208)
Supplement: Figure S2 — shows that EHMT2 activates genes mediating neuroendocrine transformation in PCa. [file crc-23-0208-s02.pdf]

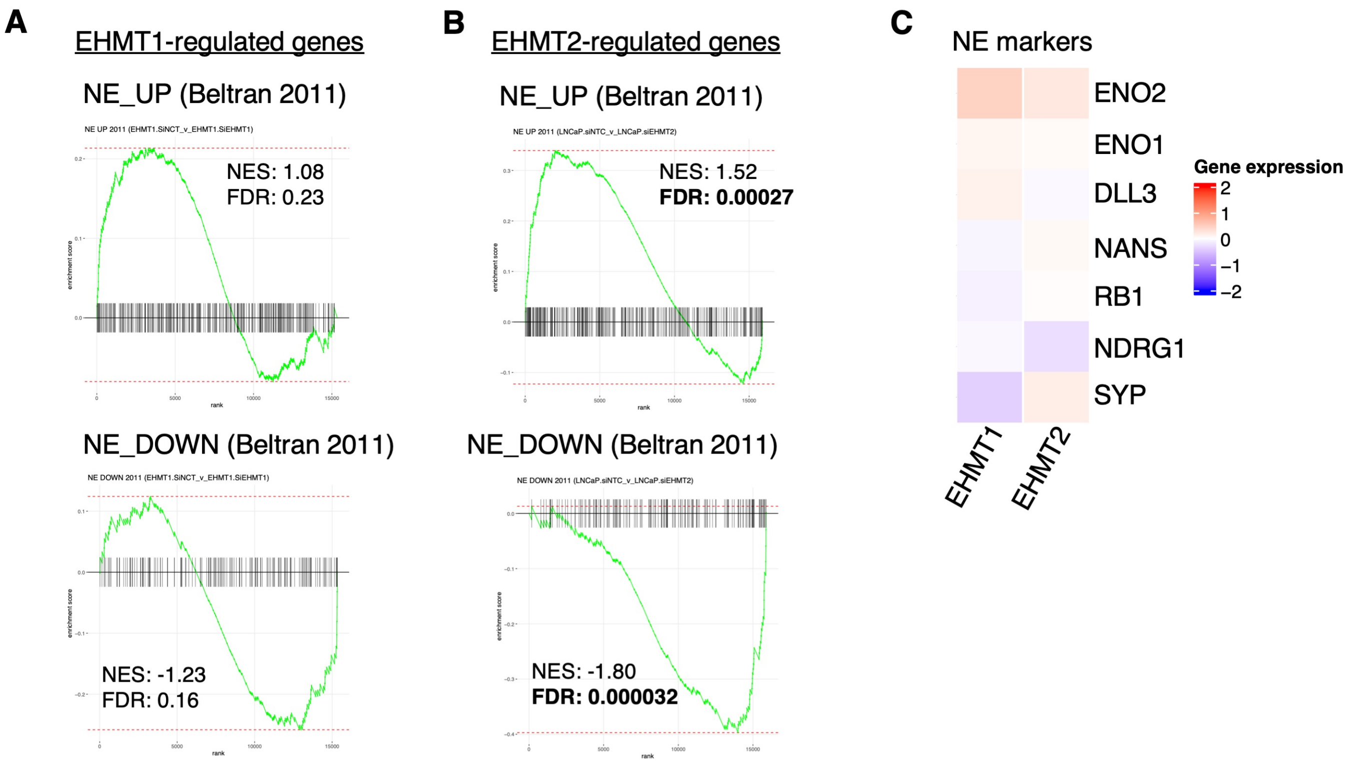

Supplementary Figure S2. EHMT2 activates genes mediating neuroendocrine transformation in PCa

(**A**, **B**) GSEA of EHMT1-regulated genes (A) and EHMT2-regulated genes (B) to assess the enrichment of previously defined gene sets upregulated (NE_UP) or downregulated (NE_DOWN) in neuroendocrine (NE) PCa (Beltran NE signatures, 2011). (**C**) Heatmap view illustrating the change of expression levels in NE markers regulated by EHMT1 or EHMT2, based on the RNA-seq analysis.
