# Supplementary material for: Demethylation of EHMT1/GLP Protein Reprograms Its Transcriptional Activity and Promotes Prostate Cancer Progression
Source: Cancer Res Commun. 2023 Aug 31;3(8):1716–30. doi: 10.1158/2767-9764.CRC-23-0208 (PMC10470473; doi:10.1158/2767-9764.CRC-23-0208)
Supplement: Figure S3 — shows that EHMT1/2 inhibitors BIX01294 and UNC0638 decrease LNCaP and PC-3 cell proliferation and migration. [file crc-23-0208-s03.pdf]

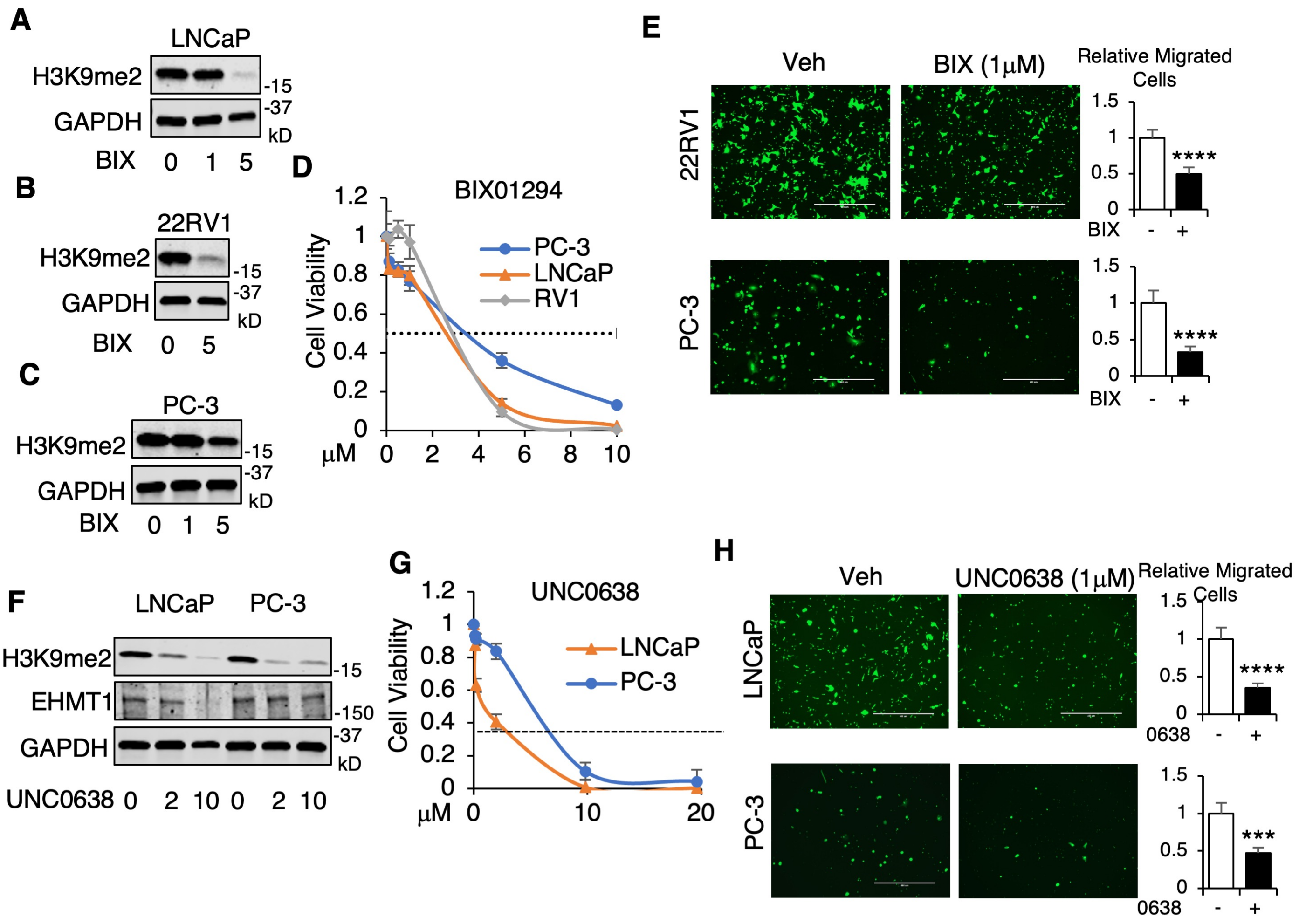

Supplementary Figure S3. EHMT1/2 inhibitors BIX01294 and UNC0638 decrease LNCaP and PC-3 cell proliferation and migration

(**A**, **B**, **C**) Immunoblotting for H3K9me2 in LNCaP (A), 22RV1 (B), or PC-3 cells (C) treated with BIX01294 (0-5μM for 3d). (**D**, **E**) Proliferation assay (D) and transwell migration assay (E) in LNCaP, PC-3, or 22RV1 cells treated with BIX01294 for 3d (0-10 μM, 3d for proliferation, 1μM, 2d for migration). (**F**, **G**, **H**) Immunoblotting for H3K9me2 and EHMT1 (F), proliferation assay (G), and transwell migration assay (H) in LNCaP or PC-3 cells treated with UNC0638 (0-20μM, 3d for proliferation; 1μM, 2d for migration).
