# Supplementary material for: Demethylation of EHMT1/GLP Protein Reprograms Its Transcriptional Activity and Promotes Prostate Cancer Progression
Source: Cancer Res Commun. 2023 Aug 31;3(8):1716–30. doi: 10.1158/2767-9764.CRC-23-0208 (PMC10470473; doi:10.1158/2767-9764.CRC-23-0208)
Supplement: Figure S4 — shows that EHMT1/2 inhibitors BIX01294 and UNC0638 cannot suppress CRPC xenograft tumors in vivo. [file crc-23-0208-s04.pdf]

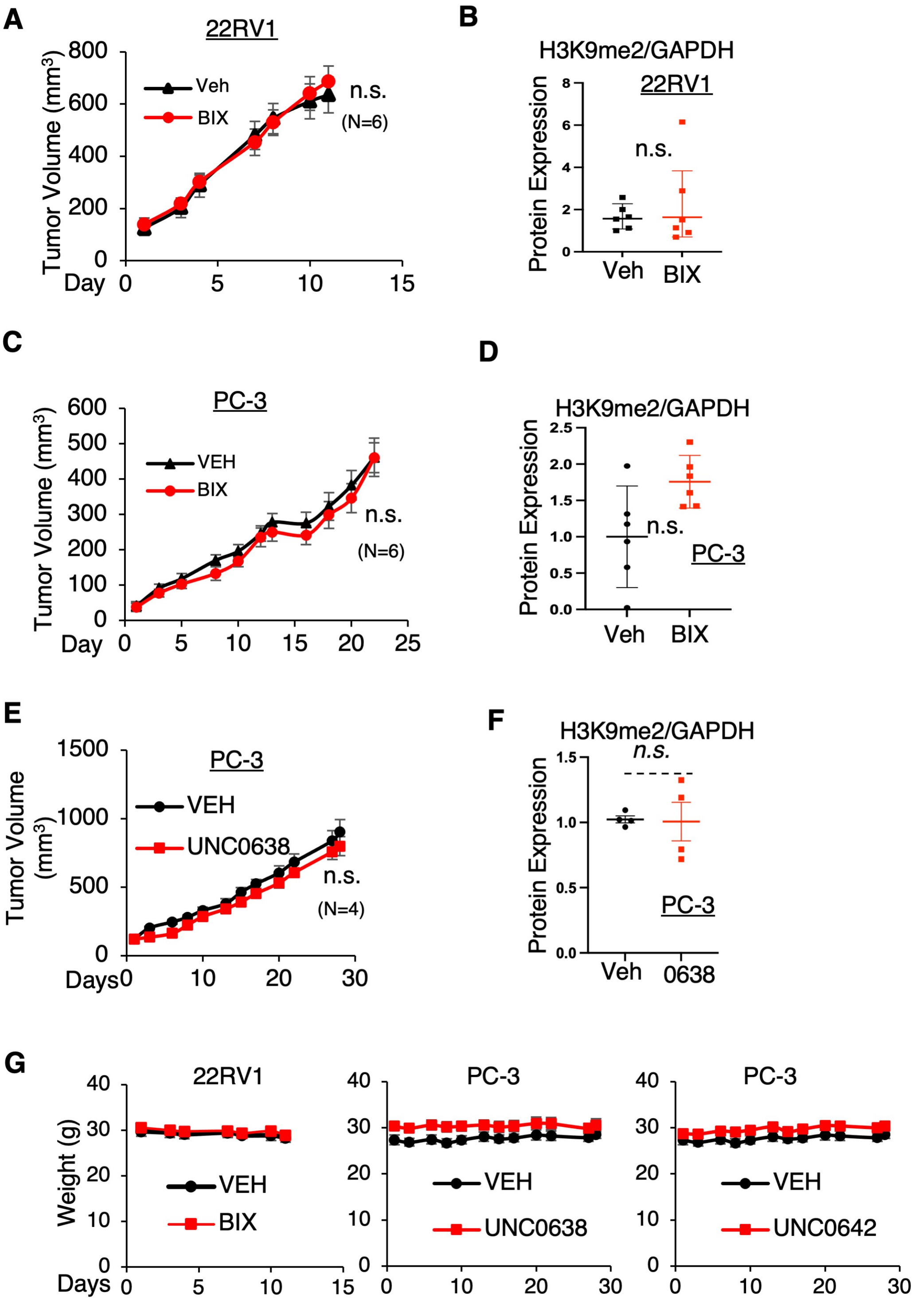

Supplementary Figure S4. EHMT1/2 inhibitors BIX01294 and UNC0638 cannot suppress CRPC xenograft tumors in vivo

(**A**, **B**, **C**, **D**) 22Rv1 cells (A) or PC-3 cells (C) were subcutaneously injected into castrated male SCID mice. Once the tumor was established, mice were treated daily with BIX01294 (30mg/kg) through oral gavage (N=6

for each model). The tumor volume was measured by caliper. Normalized H3K9me2 expression was calculated (using ImageJ) based on immunoblotting of H3K9me2 and GAPDH in CWR22-RV1 (B) model or PC-3 model (D). (**E**, **F**) PC-3 cells were subcutaneously injected into castrated male SCID mice. Mice-bearing tumors were treated daily with UNC0638 (10mg/kg) through oral gavage (N=4). The tumor volume was measured by caliper (E) and normalized H3K9me2 expression was calculated (F). (**G**) Average body weight for mice treated with BIX01294, UNC0638, or UNC0642.
