# Supplementary material for: Demethylation of EHMT1/GLP Protein Reprograms Its Transcriptional Activity and Promotes Prostate Cancer Progression
Source: Cancer Res Commun. 2023 Aug 31;3(8):1716–30. doi: 10.1158/2767-9764.CRC-23-0208 (PMC10470473; doi:10.1158/2767-9764.CRC-23-0208)
Supplement: Figure S5 — shows that K450/K451 methylations do not affect EHMT1 protein stability or nuclear localization. [file crc-23-0208-s05.pdf]

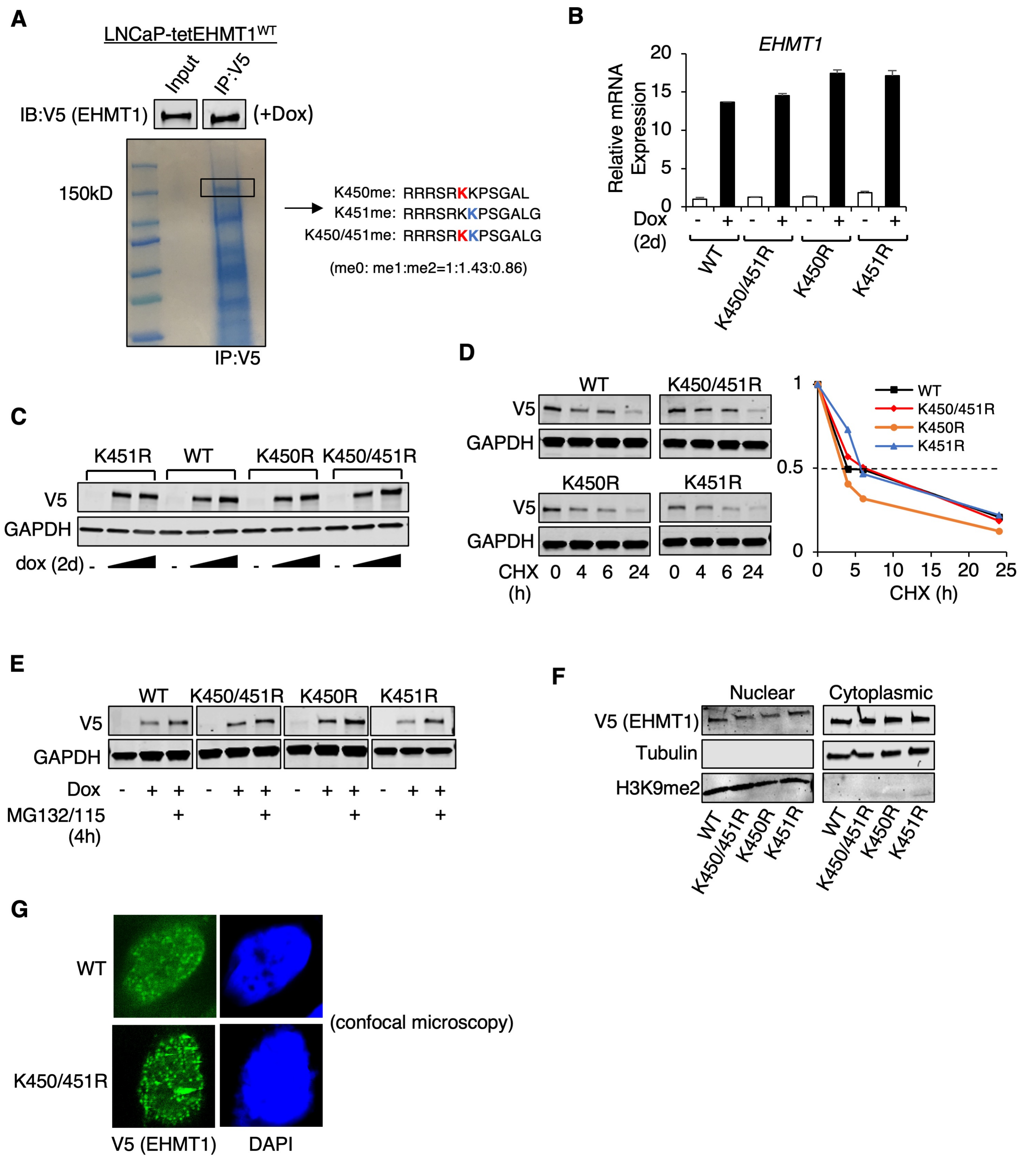

Figure S5. K450/K451 methylations do not affect EHMT1 protein stability or nuclear localization

(**A**) Immunoprecipitation for V5 (EHMT1) from LNCaP cells expressing V5-tagged doxycycline-regulated EHMT1 and stained with Coomassie blue (the 175KDa protein band was excised and digested for mass spectrometry analysis). K450 or K451 mono-methylations were the only major posttranslational modifications identified. (**B**, **C**) LNCaP stable cell lines expressing EHMT1 WT, K450R, K451R, or K450/451R mutants were established. qRT-PCR for EHMT1 mRNA expression (B) and immunoblotting for EHMT1 protein expression (C) in cells treated with doxycycline for 2 days (d). (**D**) Immunoblotting for V5 in these LNCaP stable cells (pretreated with doxycycline 2d) treated with cycloheximide (50μM, 0-24h). (**E**) Immunoblotting for V5 in stable cell lines (pre-treated with doxycycline 2d) treated with the combination of MG132/MG115 at 10μM for 4 hours (h). (**F**) Immunoblotting for indicated proteins in the nuclear extract or cytoplasmic extract of stable cell lines (pre-treated with doxycycline 2d). (**G**) Immunofluorescence staining of V5 in LNCaP stable cells expressing V5-tagged WT EHMT1 or K450/451R mutant using confocal microscopy.
