# Supplementary material for: Demethylation of EHMT1/GLP Protein Reprograms Its Transcriptional Activity and Promotes Prostate Cancer Progression
Source: Cancer Res Commun. 2023 Aug 31;3(8):1716–30. doi: 10.1158/2767-9764.CRC-23-0208 (PMC10470473; doi:10.1158/2767-9764.CRC-23-0208)
Supplement: Figure S6 — shows that Transcription profiling for WT and mutant EHMT1-overexpressing cells. [file crc-23-0208-s06.pdf]

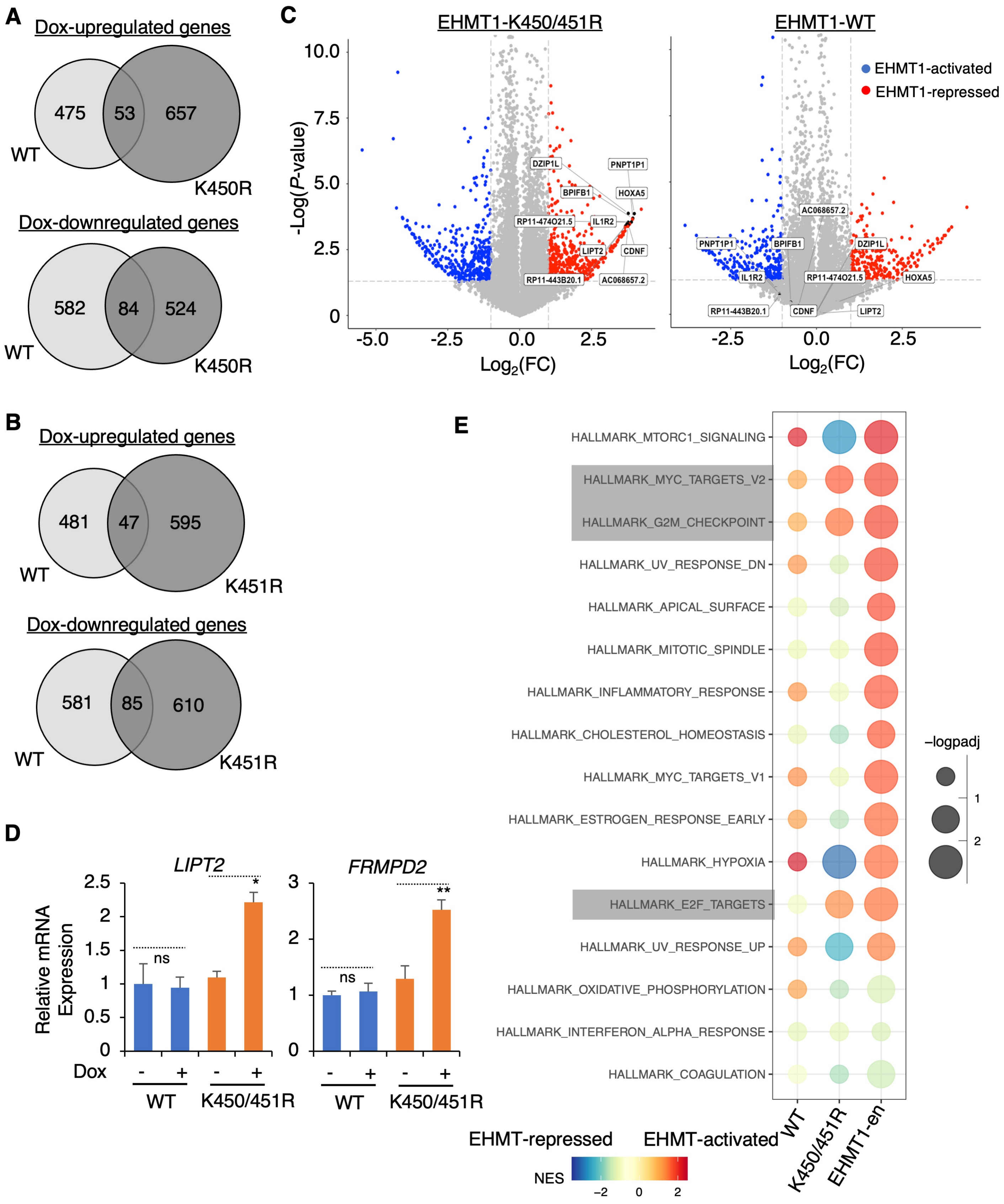
A
Dox-upregulated genes
475
53
657
WT
K450R
Dox-downregulated genes
582
84
524
WT
K450R
B
Dox-upregulated genes
481
47
595
WT
K451R
Dox-downregulated genes
581
85
610
WT
K451R
C
EHMT1-K450/451R
EHMT1-WT
EHMT1-activated
EHMT1-repressed
-Log(P-value)
Log2(FC)
DZIP1L
PNPT1P1
BPIFB1
HOXA5
RP11-474O21.5
IL1R2
LIPT2
CDNF
RP11-443B20.1
AC068657.2
D
LIPT2
FRMPD2
Relative mRNA Expression
ns
*
**
Dox
WT
K450/451R
E
HALLMARK_MTORC1_SIGNALING
HALLMARK_MYC_TARGETS_V2
HALLMARK_G2M_CHECKPOINT
HALLMARK_UV_RESPONSE_DN
HALLMARK_APICAL_SURFACE
HALLMARK_MITOTIC_SPINDLE
HALLMARK_INFLAMMATORY_RESPONSE
HALLMARK_CHOLESTEROL_HOMEOSTASIS
HALLMARK_MYC_TARGETS_V1
HALLMARK_ESTROGEN_RESPONSE_EARLY
HALLMARK_HYPOXIA
HALLMARK_E2F_TARGETS
HALLMARK_UV_RESPONSE_UP
HALLMARK_OXIDATIVE_PHOSPHORYLATION
HALLMARK_INTERFERON_ALPHA_RESPONSE
HALLMARK_COAGULATION
−logpadj
WT
K450/451R
EHMT1-en
EHMT-repressed
EHMT-activated
NES

Supplementary Figure S6. Transcription profiling for WT and mutant EHMT1-overexpressing cells

(**A**, **B**) Venn diagrams for EHMT1-WT-regulated genes versus K450R (A) or K451R-regulated (B) genes (cutoff: 2-fold, $P<0.05$). (**C**) Volcano plots for EHMT1-K450/451R and WT-regulated genes. (**D**) qRT-PCR for *LIPT2* and *FRMPD2* mRNA expression in WT or K450/451R stable cells. (**E**) GSEA for the enrichment of hallmark gene sets in endogenous EHMT1-regulated genes in comparison with WT or K450/451R-regulated genes ($P<0.05$).
