# Supplementary material for: Demethylation of EHMT1/GLP Protein Reprograms Its Transcriptional Activity and Promotes Prostate Cancer Progression
Source: Cancer Res Commun. 2023 Aug 31;3(8):1716–30. doi: 10.1158/2767-9764.CRC-23-0208 (PMC10470473; doi:10.1158/2767-9764.CRC-23-0208)
Supplement: Figure S7 — shows that K450/451R mutant-induced expression of PP1R14C can be diminished by silencing E2F1. [file crc-23-0208-s07.pdf]

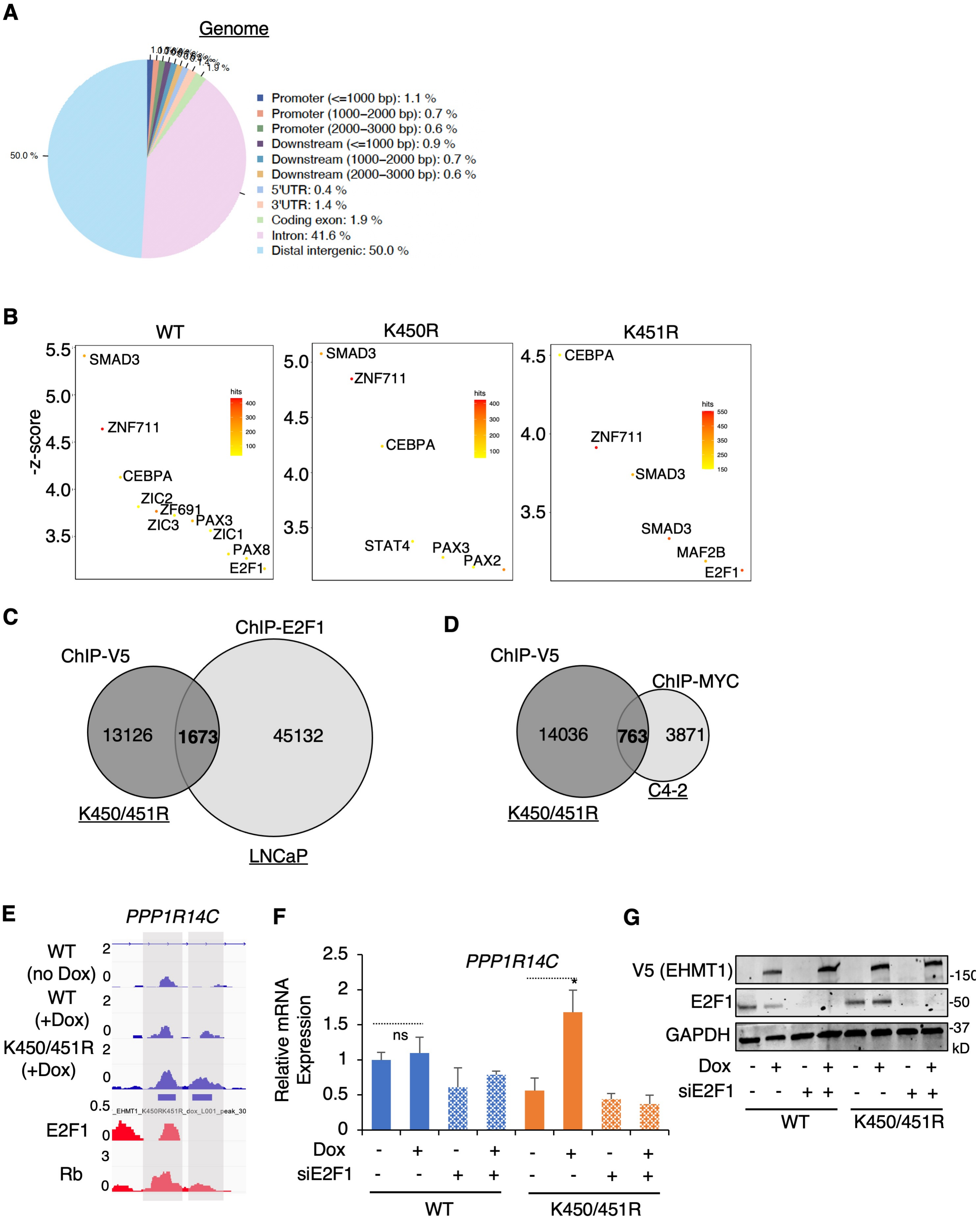
A
Genome
Promoter (<=1000 bp): 1.1 %
Promoter (1000–2000 bp): 0.7 %
Promoter (2000–3000 bp): 0.6 %
Downstream (<=1000 bp): 0.9 %
Downstream (1000–2000 bp): 0.7 %
Downstream (2000–3000 bp): 0.6 %
5'UTR: 0.4 %
3'UTR: 1.4 %
Coding exon: 1.9 %
Intron: 41.6 %
Distal intergenic: 50.0 %
50.0 %
B
WT
K450R
K451R
-z-score
hits
SMAD3
ZNF711
CEBPA
ZIC2
ZF691
ZIC3
PAX3
ZIC1
PAX8
E2F1
STAT4
PAX2
MAF2B
C
ChIP-V5
ChIP-E2F1
13126
1673
45132
K450/451R
LNCaP
D
ChIP-V5
ChIP-MYC
14036
763
3871
K450/451R
C4-2
E
PPP1R14C
WT (no Dox)
WT (+Dox)
K450/451R (+Dox)
E2F1
Rb
F
PPP1R14C
Relative mRNA Expression
ns
*
Dox
siE2F1
WT
K450/451R
G
V5 (EHMT1)
E2F1
GAPDH
-150
-50
-37
kD
Dox
siE2F1
WT
K450/451R

Supplementary Figure S7. K450/451R mutant-induced expression of *PP1R14C* can be diminished by silencing E2F1

(**A**) Chromatin distribution of regulatory regions. (**B**) Motif enrichment analyses of V5 (EHMT1) binding sites at WT, K450R, or K451R-expressing cells. (**C**, **D**) Venn diagrams for binding sites of K450/451R mutant versus published E2F1 binding sites (GSE154192) (C) or MYC binding sites (GSE179688) (D). (**E**) Genome browser view for two EHMT1-K450/451R specific binding peaks located within *PP1R14C* gene loci. (**F**, **G**) WT or K450/451R cells were pretreated with doxycycline and transfected with siNTC or siE2F1 for 3d, and then subjected to qRT-PCR for *PPP1R14C* mRNA expression (F) and immunoblotting for indicated proteins (G).
