# Supplementary material for: Demethylation of EHMT1/GLP Protein Reprograms Its Transcriptional Activity and Promotes Prostate Cancer Progression
Source: Cancer Res Commun. 2023 Aug 31;3(8):1716–30. doi: 10.1158/2767-9764.CRC-23-0208 (PMC10470473; doi:10.1158/2767-9764.CRC-23-0208)
Supplement: Figure S8 — shows that Mass-spectrometry analysis on 3xMBT-purified proteins in PCa cells treated with LSD1 inhibitor. [file crc-23-0208-s08.pdf]

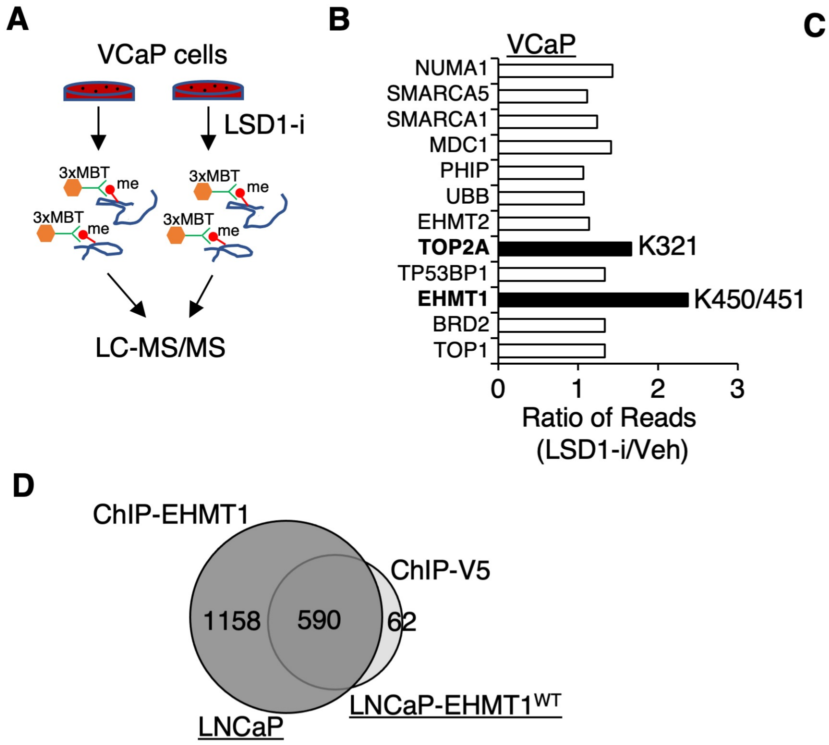


**C**

| Protein | Site |
|---|---|
| Histone 3 | K27me1/2/3 |
| Histone 3 | K36me1/2/3 |
| Histone 3 | K37me1 |
| Histone 3 | K79me1/2 |
| EEF1A2 | K55me2, K79me3, K165me2 |
| CALM1 | K80me3, K116me3<br>K117me3, K154me3, K163me3 |
| GDAP1 | K127me1, K135me3, K195me1,<br>K203me3 |
| MYO18A | K1884me1 |
| WIZ | K476me3 |
| SLC25A4 | K52me3 |
| HNRNPA1 | K161me1 |
| RSF1 | K1061me3 |
| ZNF462 | K2004me1 |
| SETD2 | K219me2 |
| EHMT1 | **K450me1, K451me1** |
| CBX3 | K143me1 |
| TOP2A | **K321me1** |

Chromatin Factors (RSF1 – TOP2A)

**Supplementary Figure S8. Mass-spectrometry analysis on 3xMBT-purified proteins in PCa cells treated with LSD1 inhibitor**

(**A**, **B**) VCaP cells treated with or without pargyline (1mM, 24h) were subjected to the 3xMBT peptide pull-down assay, followed by mass-spectrometry analysis (A). Top-ranked proteins with increased binding affinity to 3xMBT peptides were shown (B). (**C**) List of the identified methyl-lysine residues in 3xMBT interacted proteins. (**D**) The Venn diagram for ChIP-EHMT1 peaks in parental LNCaP cells in comparison with ChIP-V5 peaks in LNCaP cells expressing EHMT1-WT.
